# Supplementary material for: Short- and Long-Term Effects of UVA on Arabidopsis Are Mediated by a Novel cGMP Phosphodiesterase
Source: Curr Biol. 2019 Aug 5;29(15):2580–2585.e4. doi: 10.1016/j.cub.2019.06.071 (PMC6692503; doi:10.1016/j.cub.2019.06.071)
Supplement: Document S1. Figures S1–S4 and Table S1 [file mmc1.pdf]

Current Biology, Volume *29*

Supplemental Information

Short- and Long-Term Effects of UVA on *Arabidopsis* Are Mediated by a Novel cGMP Phosphodiesterase

Jean-Charles Isner, Vlad-Aris Olteanu, Alexander J. Hetherington, Aude Coupel-Ledru, Peng Sun, Ashley J. Pridgeon, Glyndyr S. Jones, Matthew Oates, Tom A. Williams, Frans J.M. Maathuis, Richard Kift, Ann R. Webb, Julian Gough, Keara A. Franklin, and Alistair M. Hetherington

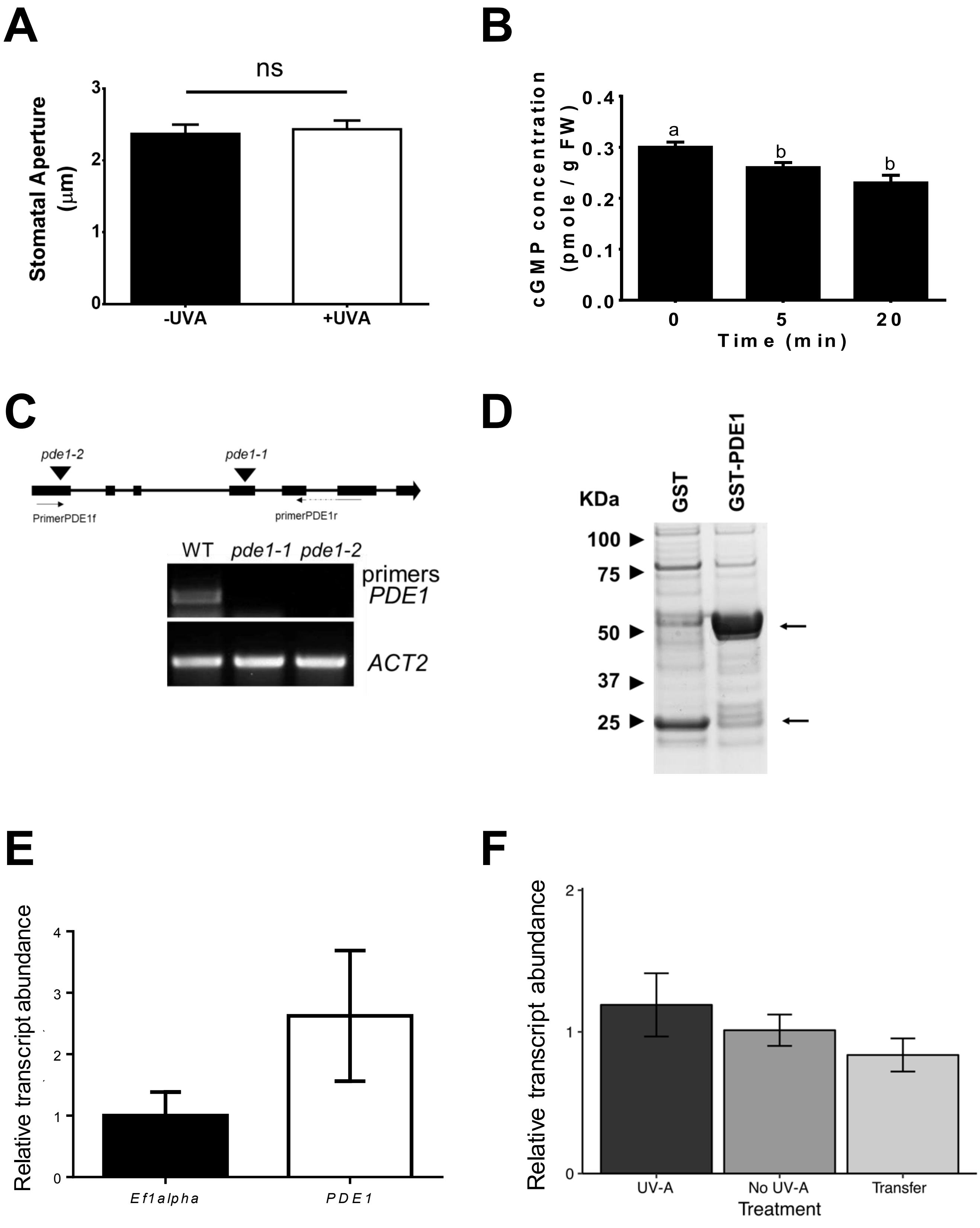
A
B
C
D
E
F
ns
Stomatal Aperture (μm)
-UVA
+UVA
cGMP concentration (pmole / g FW)
Time (min)
a
b
b
pde1-2
pde1-1
PrimerPDE1f
primerPDE1r
WT
pde1-1
pde1-2
primers PDE1
ACT2
KDa
GST
GST-PDE1
100
75
50
37
25
Relative transcript abundance
Ef1alpha
PDE1
UV-A
No UV-A
Transfer
Treatment

Figure S1: Characterisation of UVA effects on cGMP signaling pathway. Related to Figures 1, 2 and 3.

(A) UV-A radiation does not induce stomatal closure.

Isolated Arabidopsis epidermis was incubated in 10 mM MES/KOH, 50 mM KCl, pH 6.15 and illuminated with 10 µmol m^{-2} s^{-1} blue (λmax=470 nm) and 50 µmol m^{-2} s^{-1} red (λ=660 nm) for 3 hours. Subsequently, 10 µmol m^{-2} s^{-1} UV-A (λmax=380 nm) was added, or not, together with blue/red light for another 3h when stomatal apertures were measured. Data represent means (+/- SEM) of 90 stomatal aperture measurements from three replicates. Statistical analyses were performed by non-paired Student's t-test. No significant (ns) difference was observed ($p > 0.05$).

(B) UV-A induces a reduction in cGMP. Arabidopsis leaves (rosette stage) were illuminated with or without UV-A LED lights for 0, 5 and 20 min and cGMP content was determined by ELISA Exposure to UV-A (10 µmol m^{-2} s^{-1} (λmax=380 nm) results in a decrease of cGMP after 5 and 20 min. Data represent means (+/- SEM) of three replicates. Statistical analyses were performed by one-way ANOVA with Tukey posthoc analysis, and letters show significant differences at $p < 0.05$.

(C) Characterisation of cn*pde1* KO mutants. A. Exon-intron gene diagram of *AtCN-PDE1*. Exons are in black boxes. B. PDE1 was amplified by RT-PCR using gene specific primers (primerPde1f and primerPde1r, see Figure S3A) in WT. The absence of PDE1 amplification demonstrates that *pde1-1* and *pde1-2* are KO due to the presence of T-DNA. C. Sequences and domain architecture of 26 candidate PDE proteins in Arabidopsis. At1G17330 was the only one of these candidates to show a UV-A phenotype upon knockout.

(D) *In vitro* expression of GST-PDE1 in *E. coli*. *PDE1* was inserted in pGEX-6P vector. pGEX-PDE1 or empty vector was transformed into *E.coli* BL21. Expression was induced with 1mM IPTG for 4 hours at 37°C. GST-PDE1 or GST was purified using Sepharose 4G and loaded on a denaturating acrylamide gel. Gel was stained with InstantBlue™. GST-PDE1 and GST are shown by arrows at ~26KDa and 51KDa.

(E) *PDE1* is expressed in guard cells and its abundance is not affected by UV-A treatment in leaf tissue. Guard cell protoplasts were extracted from Arabidopsis leaves (see material and methods). qPCR was performed on cDNA synthetized on the RNA extracted from the guard cell protoplasts using pde1f+r primers. CN-PDE1 transcript was compared with EF1α (n=3).

(F) The transcript abundance of *PDE1* is not affected by long term or short term exposure to UV-A in leaf tissue. QPCR was performed on rosette leaves of 5 week old *Arabidopsis* Col-0 leaves that were grown under UV-A (UVA), under no UV-A (No UVA), or plants that had been grown under no UV-A and then treated with UV-A for 3 hours (Transfer). *EF1α* was used as a reference transcript. Data show the mean ± SEM (n = 3). No significant differences between the treatments was observed when analysed using a one-way ANOVA.

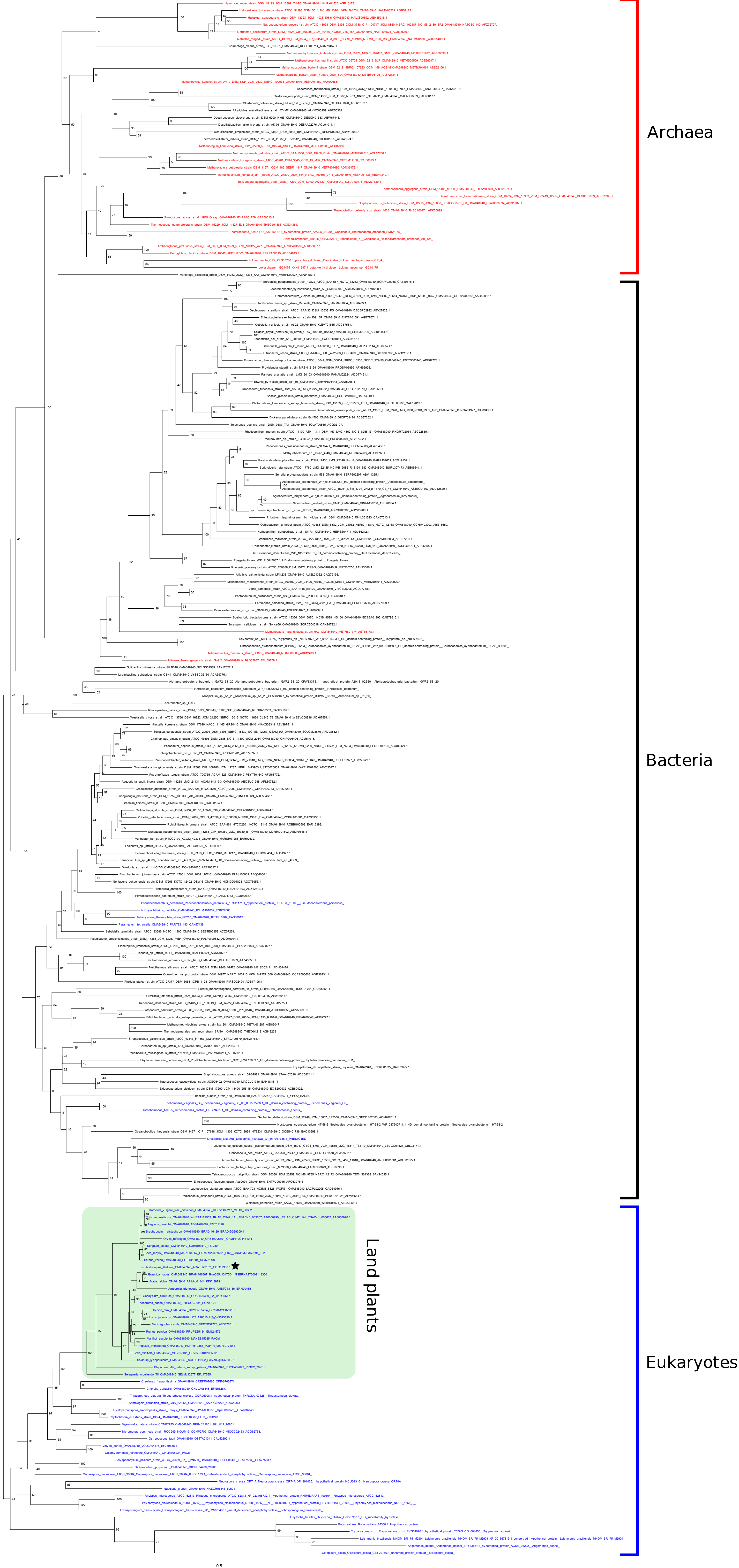
Archaea
Bacteria
Land plants
Eukaryotes
0.5

Figure S2: *CN-PDE* Tree. Related to Figure 4.

AtCN-PDE1 is part of a gene family that is conserved across the tree of life but appears to have been specifically lost in the animal lineage. Most of the eukaryotic sequences - including those from nonphotosynthetic lineages - form a monophyletic group, suggesting a single origin in the last eukaryotic common ancestor. The genes from two ciliates, *Tetrahymena* and *Paramecium*, and the excavates *Trichomonas vaginalis* and *Trichomonas foetus* appear to be recent horizontal gene acquisitions from Bacteria. The eukaryotic clade groups with Bacteria in the tree, and although members of the Asgard archaea – the closest archaeal relatives of eukaryotes – encode members of the AtPDE family, these genes are not closely related to those of eukaryotes. The phylogeny was inferred under the LG+C60+F model in IQ-Tree; branch lengths are proportional to the expected number of substitutions per site. Archaea sequences red, bacteria sequences black, eukaryotic sequences blue. Land plants highlighted with green box. At1G17330 (*CN-PDE1*) highlighted with black star.

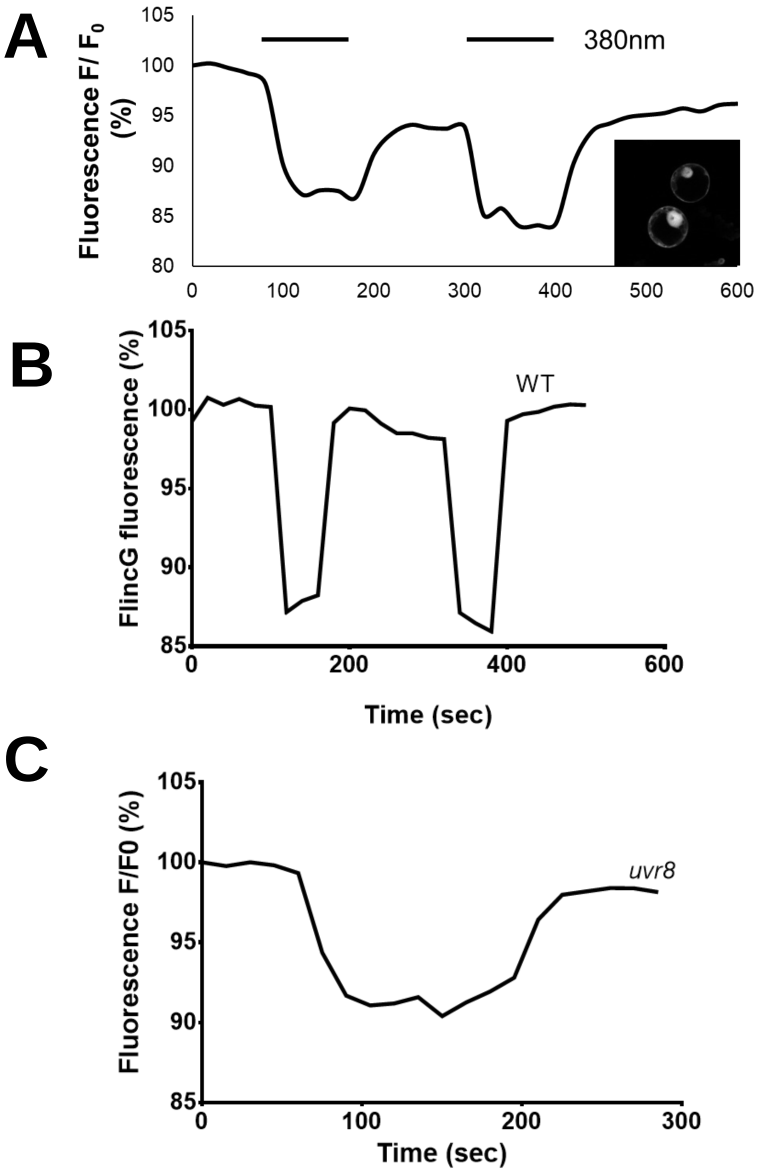

Figure S3. The UV-A signalling pathway is conserved in *Physcomitrella patens* (A) and does not involve UVR8. Related to Figure 1.

(A) Protoplasts of the moss *Physcomitrella patens* were isolated from protonemal tissue and transformed with δ-FlincG plasmid. Protoplasts were incubated 12h to allow δ-FlincG expression and the effect of UV-A on the fluorescence was monitored as described previously. The speed and the intensity of the decrease of δ-FlincG fluorescence upon UV-A illumination was similar to that observed in Arabidopsis. The graph shown is representative of 5 experiments.

(B-C) *uvr8* exhibits wild type UV-A-induced reduction in δ-FlincG fluorescence. Mesophyll protoplasts extracted from *uvr8* were transiently transformed with δ-FlincG plasmid. Protoplasts were incubated 12h to allow δ-FlincG expression and the effect of UVA on the fluorescence was monitored as described previously See methods). *uvr8* (C) showed a similar response to the WT (B) when illuminated with UVA (10 µmol m^{-2} s^{-1} (λmax=380 nm). A representative graph is shown for *uvr8* mutant tested (n > 5).

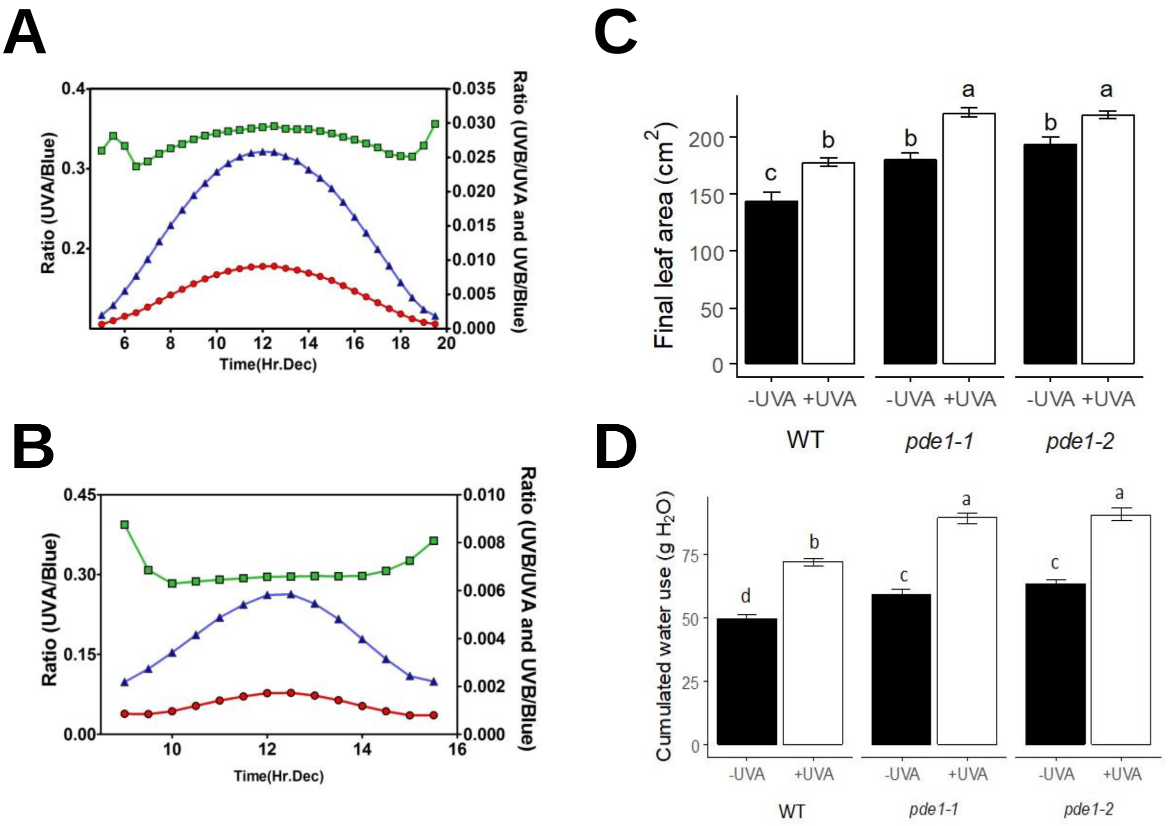

Figure S4. Diurnal cycle of UV and Blue waveband ratios in the solar spectrum and long-term UV-A effects on growth and cumulated water loss. Related to Figure 4.

(A-B) Ratios of UV-A/Blue (green squares), UV-B/Blue (red circles) and UV-B/UV-A (blue triangles) wavebands of solar radiation for cloud-free days. Measurements were made at Reading, UK (51.5N) in summer (24/7/2012) (A) and winter (13/12/2014) (B) when there were minimal disturbances due to changes in the atmosphere. Similar diurnal patterns are observed on other cloud-free days. The wavebands are the unweighted integrals of spectral measurements: UV-B = 280-315 nm; UV-A = 315-400 nm; Blue = 400-500 nm. (C-D) Long-term UV-A exposure increases growth and cumulated water loss. Arabidopsis plants were grown under light tubes supplemented with a combination of blue (λmax=470 nm) and red (λmax=660 nm) light coupled or not to UV-A light (λmax=370 nm) (as in Fig 1). For each plant, final, total plant rosette area was measured at harvest (bolting) after dissecting the plant (A). Individual pots were sealed to prevent water evaporation from the soil. From germination to bolting, cumulated water loss through transpiration was determined by daily weighing each sealed pot. The total amount of transpired water over the experiment duration is represented in (B). Data represent means (+/- SE) of 8 plants per genotype and light scenario. Statistical analyses were performed by one-way ANOVA with Tukey post-hoc analysis, and letters show significant differences at $p < 0.05$.

**Primer Name**	**Sequence**
PDE1 QPCR FP	GAAGGCGATGAAAGGAAACGA
PDE1 QPCR RP	CTGCAAGCTCCACAATTTCC
primerPde1f	TGGAAATTGTGGAGCTTGCAG
primerPde1r:	TCGTTAGTATCTTCGTCTTCTTGG
EF1α QPCR FP	TGTGCTGTTCTTATCATTGACTCC
EF1α QPCR RP	TGGCATCCATCTTGTTACAACAG
pde1GstBamHI_F	AAAGGATCCATGGCGGCGAAGACGATGA
pde1GstXhoI_R	AAACTCGAGTCAAGTTGACCCATCCCACT

Table S1. Primer Sequences. Related to STAR Methods.
