# Supplementary material for: Short- and Long-Term Effects of UVA on Arabidopsis Are Mediated by a Novel cGMP Phosphodiesterase
Source: Curr Biol. 2019 Aug 5;29(15):2580–2585.e4. doi: 10.1016/j.cub.2019.06.071 (PMC6692503; doi:10.1016/j.cub.2019.06.071)
Supplement: Document S2. Article plus Supplemental Information [file mmc3.pdf]

Current Biology

Report

Short- and Long-Term Effects of UVA on *Arabidopsis* Are Mediated by a Novel cGMP Phosphodiesterase

Highlights

- UVA inhibits stomatal opening, and this requires a reduction in the cytosolic cGMP
- UVA-induced decrease in cGMP was caused by a cGMP phosphodiesterase (*AtcnPDE1*)
- UVA radiation increased growth and decreased water use efficiency
- *cnPDE1* is ancient and has been lost from animal lineages

Authors

Jean-Charles Isner, Vlad-Aris Olteanu, Alexander J. Hetherington, ..., Julian Gough, Keara A. Franklin, Alistair M. Hetherington

Correspondence

alistair.hetherington@bristol.ac.uk

In Brief

Isner et al. show that UVA radiation inhibits stomatal opening in *Arabidopsis*. This requires a reduction in cytosolic cGMP, mediated by a cGMP-activated phosphodiesterase (*AtCN-PDE1*) that is conserved across the tree of life, except in metazoans, where it has been lost. They also show that UVA increases growth and decreases water use efficiency.

Isner et al., 2019, Current Biology *29*, 2580–2585
August 5, 2019 © 2019 The Authors. Published by Elsevier Ltd.
https://doi.org/10.1016/j.cub.2019.06.071

CellPress
Current Biology
Report

Short- and Long-Term Effects of UVA on *Arabidopsis* Are Mediated by a Novel cGMP Phosphodiesterase

Jean-Charles Isner,[1] Vlad-Aris Olteanu,[2] Alexander J. Hetherington,[3] Aude Coupel-Ledru,[1] Peng Sun,[1] Ashley J. Pridgeon,[1] Glyndyr S. Jones,[1] Matthew Oates,[2] Tom A. Williams,[1] Frans J.M. Maathuis,[4] Richard Kift,[5] Ann R. Webb,[5] Julian Gough,[6] Keara A. Franklin,[1] and Alistair M. Hetherington[1,7,*]
[1]School of Biological Sciences, Life Sciences Building, University of Bristol, 24 Tyndall Avenue, Bristol BS8 1TH, UK
[2]Department of Computer Science, Merchant Venturers Building, University of Bristol, Woodland Road, Bristol BS8 1UB, UK
[3]Department of Plant Sciences, University of Oxford, South Parks Road, Oxford OX1 3RB, UK
[4]Department of Biology, University of York, York YO10 5DD, UK
[5]School of Earth and Environmental Sciences, University of Manchester, Simon Building, Oxford Road, Manchester M13 9PL, UK
[6]MRC Laboratory of Molecular Biology, Francis Crick Avenue, Cambridge Biomedical Campus, Cambridge CB2 0QU, UK
[7]Lead Contact
*Correspondence: alistair.hetherington@bristol.ac.uk
https://doi.org/10.1016/j.cub.2019.06.071

SUMMARY

Although UVA radiation (315–400 nm) represents 95% of the UV radiation reaching the earth's surface, surprisingly little is known about its effects on plants [1]. We show that in *Arabidopsis*, short-term exposure to UVA inhibits the opening of stomata, and this requires a reduction in the cytosolic level of cGMP. This process is independent of UVR8, the UVB receptor. A cGMP-activated phosphodiesterase (*AtCN-PDE1*) was responsible for the UVA-induced decrease in cGMP in *Arabidopsis*. AtCN-PDE1-like proteins form a clade within the large HD-domain/PDEase-like protein superfamily, but no eukaryotic members of this subfamily have been functionally characterized. These genes have been lost from the genomes of metazoans but are otherwise conserved as single-copy genes across the tree of life. In longer-term experiments, UVA radiation increased growth and decreased water-use efficiency. These experiments revealed that *PDE1* is also a negative regulator of growth. As the *PDE1* gene is ancient and not represented in animal lineages, it is likely that at least one element of cGMP signaling in plants has evolved differently to the system present in metazoans.

RESULTS

In contrast to the well-known effects of visible and UVB radiation [2], much less is known about the effects of UVA radiation (315–400 nm) on plants. UVA has been reported to affect root and shoot biomass, the accumulation of phenolics, and photosynthetic rates. However, the effects are variable, ranging from positive through no effect to negative [1–3]. To help define the effects of UVA on plants, stomata were selected for investigation [4]. Because stomata, in the natural environment, would not experience UVA irradiation in isolation, the effect of UVA in combination with blue and red light on stomatal aperture was investigated. Exposure to 10 μmol m^{-2} sec^{-1} UVA radiation resulted in a reduction in blue- and red-light-induced stomatal opening (Figure 1A). The inhibitory effect was greater after 3 h exposure to UVA than at 6 h, suggesting that with time, the extent of the negative effect on blue- and red-light-induced opening decreased. In contrast, UVA failed to induce closure in stomata that had previously been opened by exposure to red and blue light (Figure S1A). These results show that short-term (3–6 h) exposure to UVA inhibits blue- and red-light-induced opening, but the effect decreases with time. However, UVA does not promote stomatal closure.

To learn more about the signaling pathway underlying this response, the intracellular second messenger, cyclic guanosine monophosphate (cGMP), was selected for investigation. This was because cyclic nucleotide-gated ion channels are expressed in guard cells [5–7], and cGMP and its nitrated relative, 8-nitro-cGMP, are implicated in the control of stomatal aperture [8–10]. Illumination of either mesophyll protoplasts (Figure 1B) or guard cells in isolated epidermes (Figure 1C) expressing the cGMP fluorescent reporter, δ-FlincG [11], with 10 μmol m^{-2} sec^{-1} UVA resulted in a reduction in the UVA-induced change in δ-FlincG fluorescence. Figure 1B also shows that when UVA illumination was withdrawn, δ-FlincG fluorescence rapidly returned to pre-UVA illumination levels. Unlike UVA, neither red nor far-red light induced reductions in δ-FlincG fluorescence (Figure 1D). As an independent confirmation that UVA induces a reduction in cGMP, ELISA was used (Figure S1B). Together, these results show that UVA induces a reversible reduction in cytosolic cGMP levels in guard and mesophyll cells.

To test whether UVA acts on cGMP biosynthesis or degradation, mesophyll protoplasts were incubated in the presence of the guanylyl cyclase inhibitor LY-83583 (50 μM) [12] for 10 min prior to exposure to pulses of UVA (10 μmol m^{-2} s^{-1}). UVA-induced changes in fluorescence (Figure 2A) ranged from 15% reduction in the absence of the inhibitor to a 5% reduction in the presence of LY-83583. The addition of IBMX (50 μM), a

 © 2019 The Authors. Published by Elsevier Ltd.
This is an open access article under the CC BY license (http://creativecommons.org/licenses/by/4.0/).

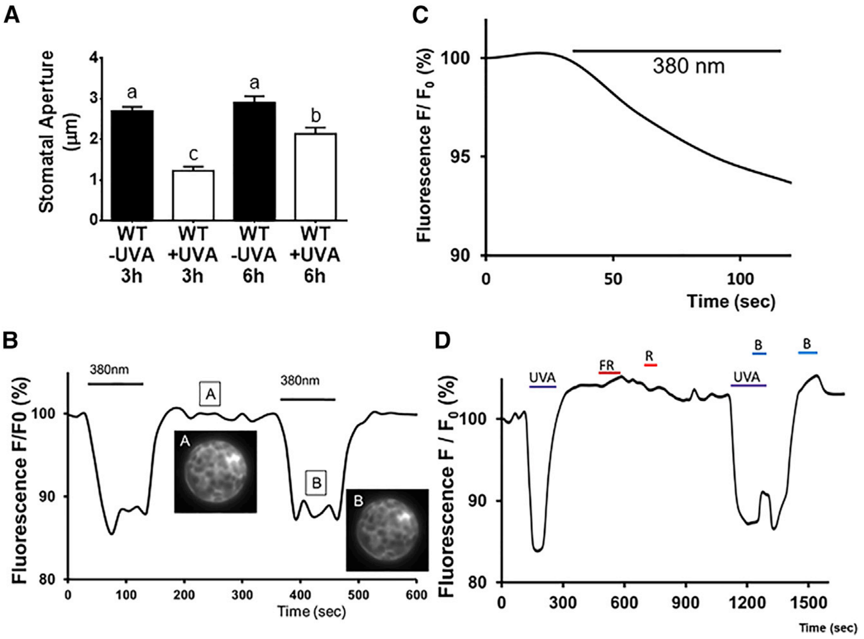

Figure 1. Effects of UVA Radiation on Stomatal Opening and δ-FlincG Fluorescence

(A) UVA radiation inhibits blue- and red-light-induced stomatal opening. Isolated *Arabidopsis* epidermis were illuminated with a combination of 10 μmol m^{-2} s^{-1} blue (λmax = 470 nm) and 50 μmol m^{-2} s^{-1} red (λmax = 660 nm) light in the presence or absence of 10 μmol m^{-1} s^{-1} UVA (λmax = 380 nm) in 10 mM MES/KOH, 50 mM KCl, pH 6.15. Stomatal apertures were measured after 3 h and 6 h illumination. Data represent means (+/− SEM) of 90 stomatal aperture measurements from three replicates. Statistical analyses were performed by one-way ANOVA with Tukey post hoc analysis, and letters show significant differences at $p < 0.05$.

(B) A representative graph (n = 50) of the reversible decrease of δ-FlincG fluorescence observed in mesophyll protoplasts when illuminated with 10 μmol m^{-1} s^{-1} UVA (λmax = 380 nm). In the presence of UVA, the fluorescence decreases within seconds and reaches a plateau. In the absence of UVA, the fluorescence rapidly returns to its initial level. The lines labeled 380 nm represent the length of time the protoplasts were illuminated with UVA.

(C) A representative measurement (n = 5) of δ-FlincG fluorescence from a guard cell on an isolated epidermal strip illuminated with UVA radiation (10 μmol m^{-1} s^{-1} [λmax = 380 nm]). The line labeled 380 nm represents the length of time the epidermal strip was illuminated with UVA.

(D) Effect of light quality on δ-FlincG fluorescence. Fluorescence of mesophyll protoplasts from plant expressing δ-FlincG was measured when illuminated with UVA (λmax = 380 nm, 10 μmol m^{-1} s^{-1}), blue (λmax = 470 nm, 20 μmol m^{-1} s^{-1}), red (λmax = 680 nm, 15 μmol m^{-1} s^{-1}) or far-red (λmax = 730 nm, 5 μmol m^{-1} s^{-1}) light. Far-red and red light did not have a significant effect on the fluorescence levels. UVA decreases the fluorescence by 15%, and blue increases it by 2.5% (n = 3, $p < 0.05$). The lines represent the length of time the protoplasts were illuminated with UVA, far-red (FR), red (R), UVA, and blue (B) together or blue alone. The graph shown is representative of 3 independent experiments.

See also Figures S1 and S3.

general inhibitor of cyclic nucleotide phosphodiesterases [13], resulted in no UVA-induced reduction in fluorescence (Figure 2B). These results suggested that the UVA-induced reduction in cGMP was mediated by the combined action of IBMX-sensitive cGMP-PDE activity and LY-83583-sensitive guanyl cyclase activity. Since the greater effect was due to putative cGMP-PDE activity, the effect of IBMX on the ability of UVA to inhibit blue- and red-light-induced stomatal opening was investigated. The data in Figure 2C show that IBMX abolished the UVA inhibition of blue- and red-light-induced stomatal opening, consistent with a role for cGMP-PDE activity in this response. To understand the mechanism underlying the effect of UVA on stomata in more detail, the possible role of cGMP-PDE activity in this response was investigated.

Although there is a report of an adenylyl cyclase with a phosphodiesterase domain in Marchantia, the gene does not appear to be present in gymnosperms or angiosperms [14]. This, coupled with the lack of cGMP-PDE-encoding genes [5, 15] in *Arabidopsis* or any other vascular plant, prompted the use of bespoke hidden Markov models, large-scale cross-species comparison methods, and gene-model-free searches to identify candidate cGMP-PDE sequences. This strategy resulted in the identification of 26 putative *cGMP-PDEs* in the genome of *Arabidopsis thaliana* (see Data S1 for more information). Mutants in each of the putative *A. thaliana* candidate genes were obtained from public collections. Mesophyll protoplasts were extracted from these mutants and were transiently transformed with δ-FlincG. Subsequently, the response to UVA illumination was monitored. UVA induced reductions in fluorescence, similar to WT, in all candidate *cGMP-PDE* mutants except for two allelic lines of *At1G17330* (Figures 3A and S1C). Henceforth, *At1G17330* is referred to as *CN-PDE1*, for cyclic nucleotide-dependent phosphodiesterase 1. *CN-PDE1* transcripts were not detectable in either *cn-pde1-1* or *cn-pde1-2*, indicating that they are knock-out mutants (Figure S1C).

To test whether *CN-PDE1* encodes a protein with cGMP-PDE activity, the gene was cloned, the recombinant GST::CN-PDE1 fusion protein was expressed (Figure S1D), and its cGMP phosphodiesterase activity was assayed. This revealed that CN-PDE1 exhibited cGMP-stimulated PDE activity (Vmax and Km were 58.22 pmole min^{-1} μg $protein^{-1}$ and 0.259 μM, respectively), and it was inhibited by 50 μM IBMX (Figures 3B and 3C). This enzyme activity is about 1,000-fold higher than cGMP PDE activity reported in crude preparations from *Arabidopsis* (1.25 nmol min^{-1} mg^{-1} protein) [9] but is lower than that obtained from purified mammalian CN-PDE enzymes [16]. Figure S1F shows that *CN-PDE1* is expressed in guard cells, and its expression is not altered in the presence of UVA. Figure 3D shows that, in contrast to wild-type, UVA-induced inhibition of stomatal opening by red and blue light was abolished in *cn-pde1-1* and *cn-pde1-2*. Together, these data show that *CN-PDE1* encodes an enzyme with cGMP-PDE activity that is required for UVA-induced reduction in cGMP levels, and UVA induced inhibition of blue- and red-light-mediated stomatal opening.

While cGMP is present in plant cells, and there have been reports of guanylyl cyclase activity in plants [15, 17], the

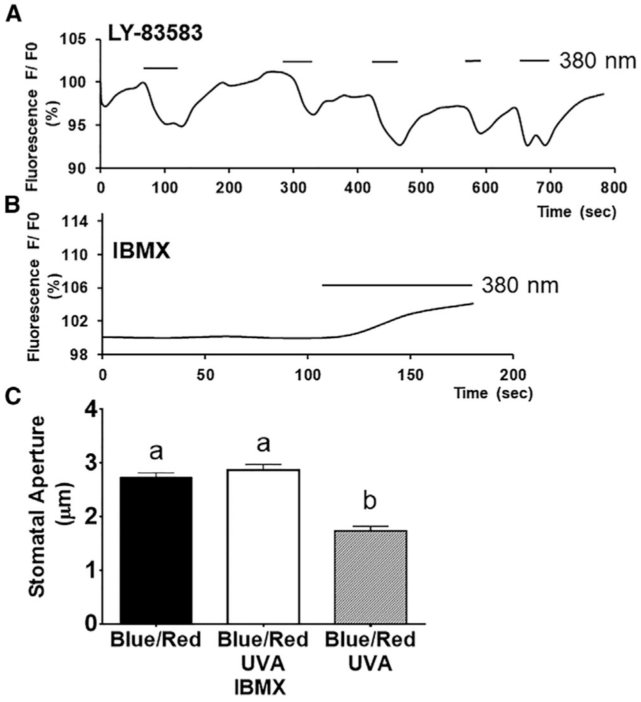

Figure 2. Effect of Cyclic Nucleotide Metabolism Inhibitors on UVA-Induced Responses

(A) The guanylyl cyclase inhibitor LY-83583 partially interferes with UVA-induced reductions in δ-FlincG fluorescence. Mesophyll protoplasts were treated with 50 μM LY-83583 for 10 min, followed by pulses of UVA (10 μmol m^{-2} s^{-1} [λmax = 380 nm]), and fluorescence was recorded (representative graph of 3 experiments).

(B) The phosphodiesterase inhibitor IBMX inhibits UVA-induced reductions in fluorescence (representative graph of 3 experiments). Mesophyll protoplasts were incubated with 50 μM IBMX for 10 min and δ-FlincG fluorescence was subsequently recorded in the presence of UVA (10 μmol m^{-2} s^{-1} [λmax = 380 nm]).

(C) IBMX prevents UVA-induced inhibition of blue- and red-light-induced stomatal opening. *Arabidopsis* epidermis was incubated in 10 mM MES/KOH, 50 mM KCl, pH 6.15 and illuminated with 10 μmol m^{-2} s^{-1} blue (λmax = 470 nm) and 50 μmol m^{-2} s^{-1} red (λmax = 660 nm) in the presence or absence of 10 μmol m^{-1} s^{-1} UVA (λmax = 380 nm) and in the presence or absence of 50 μM IBMX. Stomatal apertures were measured after 3 h. Data represent means (+/− SEM) of 90 stomatal aperture measurements from three replicates. Statistical analyses were performed by one-way ANOVA with Tukey post hoc analysis, and letters show significant differences at $p < 0.05$.

See also Figures S1 and S3.

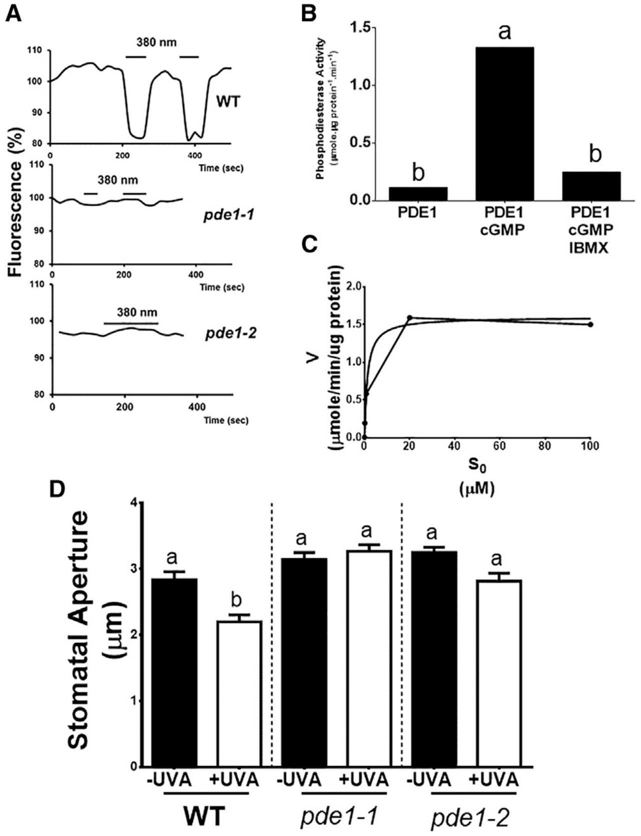

Figure 3. *AtPDE1* Encodes a Phosphodiesterase Activated by cGMP and Inhibited by IBMX

(A) Mesophyll protoplasts were extracted from WT, *pde1-1* and *pde1-2* leaves and transiently transformed with δ-FlincG. UVA illumination (10 μmol m^{-2} s^{-1} [λmax = 380 nm]) induced a significant reduction in δ-FlincG fluorescence in WT, but no significant decrease was observed in either *pde1-1* or *pde1-2* (representative plot out of 3 experiments).

(B) cGMP activity of PDE1. PDE1 was expressed *in vitro*, and its cGMP activity was measured. PDE1 activity was stimulated by cGMP (50 μM) and inhibited by 50 μM IBMX. Data represent means. Statistical analyses were performed by one-way ANOVA with Tukey post hoc analysis. Letters show significant differences at $p < 0.05$.

(C) Michaelis-Menten plot of the phoshopdiesterase activity of PDE1. Vmax and Km observed were 58.22 pmole min^{-1} μg $protein^{-1}$ and 0.2587 μM, respectively.

(D) *pde1-1 and pde1-2* do not exhibit UVA-induced inhibition of blue- and red-light-induced stomatal opening. Isolated epidermes were incubated in buffer 10 mM MES/KOH, 50 mM KCl, pH 6.15 and illuminated with 10 μmol m^{-2} s^{-1} blue (λmax = 470 nm) and 50 μmol m^{-2} s^{-1} red (λmax = 660 nm) in the presence or absence of 10 μmol m^{-2} s^{-1} UVA (λmax = 380 nm). Stomatal apertures were measured after 3 h illumination. Data represent means (+/− SEM) of 90 stomatal aperture measurements from three replicates. Statistical analyses were performed by one-way ANOVA with Tukey post hoc analysis, and letters show significant differences at $p < 0.05$.

See also Figures S1 and S3.

Arabidopsis genome apparently does not to contain a gene homologous to protein kinase G [18, 19], suggesting that there are likely to be difference between the way that cGMP signaling operates in plant and mammalian cells [20]. To gain insights into the evolution of cGMP signaling in plants, a phylogenetic investigation into the *AtCN-PDE1* gene was conducted. Examination of the protein structure of AtCN-PDE1 reveals that it is a member of the HD-domain/PDEase-like protein superfamily. Orthologs of *AtCN-PDE* are conserved across the tree of life. It is widespread in bacteria and archaea, and it is present in all the major eukaryotic lineages (Excavata, SAR, Archaeplastida, and Amorphea) but has been lost in animals after the divergence from their closest unicellular relatives (Figures 4A and S2). The *Bacillus subtilus* and *Escherichia coli* orthologs, YpgQ and YedJ, have been structurally and biochemically characterized as Mn^{2+}-dependent (d)NTP pyrophosphohydrolases [21]. This (d) NTP pyrophosphohydrolase activity appears to be conserved among orthologs from a diverse range of Bacteria [21], although substrate specificity varies. The cellular role of the protein in Bacteria is unclear, but gene expression data and gene neighborhood analysis suggest that it may be involved in DNA repair

or the maintenance of DNA replication fidelity, perhaps via the hydrolysis of excess or damaged (d)NTPs [21]. *AtCN-PDE* orthologs are highly conserved as single-copy genes in all plant genomes examined (the only exception was the genome of *Pinus taeda*, although *AtCN-PDE* orthologs were identified in transcriptomes of other pine species [22], so the absence in the genome may be due to incomplete sampling or species-specific loss). This high conservation as a single-copy gene suggests that the protein product of this gene is important to the land plant lineage. However, AtCN-PDE1-like proteins are notably absent from the genomes of metazoans, suggesting first that they have been lost in animals and second that at least one plant PDE protein has evolved independently in plants and animals.

To investigate whether the UVA-mediated reduction in δ-FlincG fluorescence is also evolutionarily conserved, the investigation was expanded to include the moss *Physcomitrella patens* as a representative of the bryophytes, which may be the most distantly related lineage of land plants compared to *Arabidopsis* [23]. *P. patens* protoplasts were extracted and transformed with δ-FlincG, and the effect of UVA on the δ-FlincG fluorescence was investigated. The data in Figure S3A show that in *Physcomitrella patens*, just as in *Arabidopsis*, UVA induces a decrease in cGMP. These data suggest that at least some aspects of the UVA response are evolutionarily conserved.

DISCUSSION

The data presented here reveal an apparently evolutionarily conserved response to UVA radiation that is likely mediated, at least in part, by a cGMP-PDE, not present in metazoans. This raises four immediate questions: first, is the UVA effect on cGMP levels present in evolutionarily more basal plant lineages? As UVA induces a decrease in δ-FlincG fluorescence in the moss *Physcomitella patens,* it is possible to conclude that the ability to respond to UVA is likely conserved over the approximately 500 million years of land plant evolution [24]. Second, is the UVB photoreceptor, UVR8, which is known to be involved in UVB-promoted stomatal closure in *Arabidopsis* [25], involved in the UVA response? Because the *uvr8* mutant exhibits wild-type UVA-induced reductions in δ-FlincG fluorescence (Figures S3B and S3C), it is apparent that UVA signaling does not require the UVB receptor. Additional questions are, what are the likely downstream targets of cGMP in plants, and second, how significant are the effects of UVA radiation on plant growth and water relations? While higher plants apparently lack a key downstream mediator of cGMP signaling in animals, protein kinase G [19], they do possess cyclic nucleotide-gated ion channels that are sensitive to changes in cGMP and are known to be important

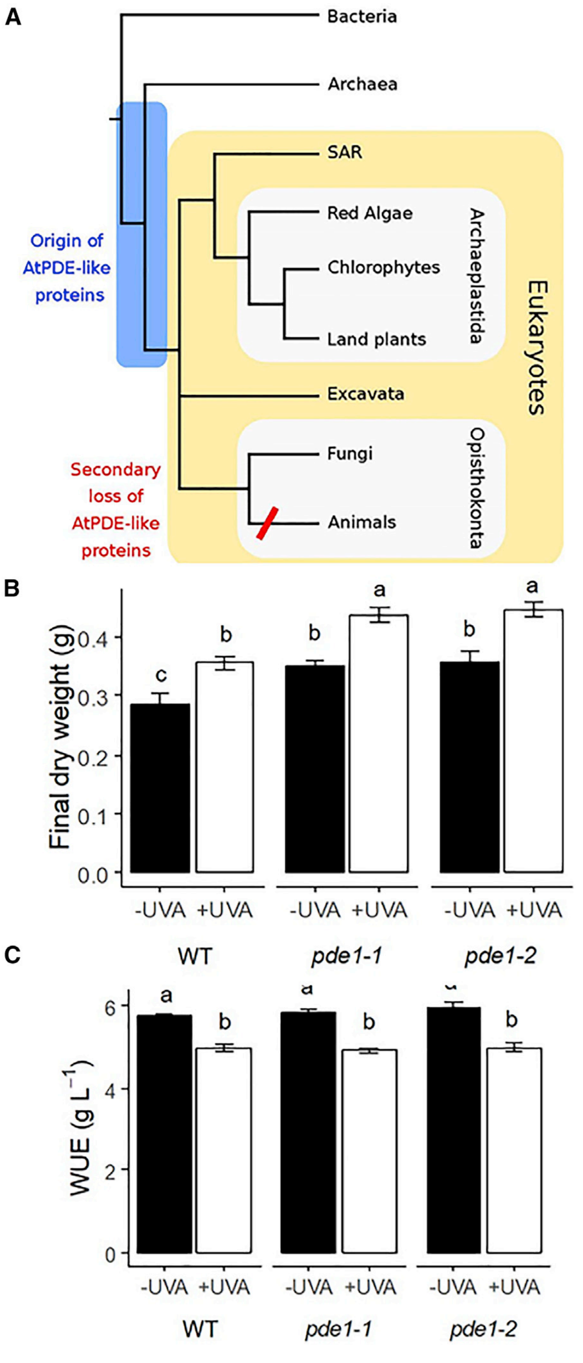

Figure 4. Evolution of AtPDE-Like Proteins and Effect of UVA on Growth and WUE

(A) Cartoon species tree of the major groups of life highlighting the origin and later secondary loss of AtPDE-like proteins. Based on the widespread presence of AtPDE-like proteins in bacteria, archaea, and eukaryotes, we predict an origin of AtPDE-like proteins prior to the last eukaryotic common ancestor highlighted with blue box. AtPDE-like proteins were then conserved in the major groups of eukaryotes before being secondarily lost in the animal stem lineage prior to the diversification of the modern animal groups.

(B and C) Long-term UVA exposure enhances growth and does not affect water-use efficiency. *Arabidopsis* plants were grown under light tubes supplemented with a combination of blue (λmax = 470 nm) and red (λmax = 660 nm) light coupled or not to UVA light (λmax = 370 nm) (as in Figure 1). All conditions other than presence or absence of UVA were identical (PAR = 120μmol m^{-2} s^{-1}, temperature 22/20°C [day/night], relative humidity 70%, 12 h photoperiod). Final plant dry weight was measured at bolting (B). Individual pots were sealed to stop water evaporation from the soil, and each pot was weighed daily from germination until bolting to compute cumulated plant water loss through transpiration. Whole plant water-use efficiency (WUE) was calculated as the ratio between final dry weight and cumulated water loss (C). Data represent means (+/− SE) of 8 plants per genotype and light scenario. Statistical analyses were performed by one-way ANOVA with Tukey post hoc analysis, and letters show significant differences at $p < 0.05$.

See also Figure S2 and Data S1 for more information. See also Figure S4.

in guard cell turgor regulation [5, 7]. To gain an insight into the physiological significance of the results reported here, we searched our database of solar UV spectra measured in south-east England for cloudless days, and then we examined changes in the ratio of UVA to blue light on such days (Figure S4). The highest ratios of UVA to blue radiation are encountered when the sun is at its midday zenith, or on the horizon, although radiation at all wavelengths is minimal at this time. Because the data in Figure 1A show that UVA radiation inhibits blue-light-induced stomatal opening, it is possible to conclude that short-term exposure to UVA radiation acts as a ''brake'' on blue-light-induced stomatal opening (assuming that the LED-based λmax 380 nm radiation used here is representative of the response to the full UVA wave-band, 315–400 nm). In order to investigate the physiological significance of UVA radiation to the plant over the longer term, growth and water use efficiency (WUE) were measured on plants grown with or without UVA. Cumulated plant water use and daily increase in rosette area were measured over a period of 22 days (from 16 days after sowing until bolting), and final plant biomass was measured at harvest. Growth (final dry weight and leaf area, Figures 4B, 4C, and 4D) was significantly enhanced, irrespective of genotype, in the presence of UVA. In the two mutant alleles of *pde1*, growth was significantly greater than in WT (Figure 4B). These data show that UVA is a positive regulator of growth, while the *PDE1* gene is a negative regulator. The data in Figure 4C show that WUE significantly decreases in all genotypes when grown in the presence of UVA, and this reflects the increase in cumulative water loss seen in all genotypes (Figure S4D). Together, our data show that in the short term, measured in hours, UVA radiation acts as a brake on blue- and red-light-simulated stomatal opening. However, in the longer term, measured over days and weeks, these effects are superseded, because UVA induces increased transpirational water loss, increased growth, and an overall decrease in WUE. Whether an alteration in stomatal density, induced by growth in the presence of UVA, contributes to the decrease in WUE remains to be examined. Under these long-term growth conditions the *PDE1* gene acts as negative regulator of plant growth.

In conclusion, we report that the short- and long-term effects of UVA radiation on *Arabidopsis* are mediated, at least in part, by a signaling system based on cGMP and the novel cGMP-phosphodiesterase encoded by the *PDE1* gene. As this gene is ancient and not represented in animal lineages, it is likely that at least one element of cGMP signaling in plants has evolved differently to the system present in metazoans.

STAR★METHODS

Detailed methods are provided in the online version of this paper and include the following:

- KEY RESOURCES TABLE
- LEAD CONTACT AND MATERIALS AVAILABILITY
  - ○ Short-term response to UVA: experimental model and subject details
- METHOD DETAILS
  - ○ Stomatal aperture measurement
  - ○ Fluorescence microscopy
  - ○ ELISA cGMP immunodetection
  - ○ *In vitro* activity of PDE1
  - ○ qPCR
  - ○ Long-term response to UVA experiment
  - ○ Bioinformatic analysis
  - ○ Phylogenomic analysis
  - ○ UVA spectrum
- QUANTIFICATION AND STATISTICAL ANALYSES
  - ○ Statistical tests
- DATA AND CODE AVAILABILITY

SUPPLEMENTAL INFORMATION

Supplemental Information can be found online at https://doi.org/10.1016/j.cub.2019.06.071.

ACKNOWLEDGMENTS

A.M.H. acknowledges support from the UK Biotechnology and Biological Sciences Research Council, grant number BB/N001168/1 and the Leverhulme Trust. A.R.W. acknowledges support for UV monitoring from the Department for Environment, Food and Rural Affairs. A.C.-L. received an individual Marie Curie Fellowship (MSCA-IF H2020)

AUTHOR CONTRIBUTIONS

J.C.I. and A.M.H. conceptualized the study. Experiments and data analysis were carried out by V.-A.O., A.J.H., M.O., P.S., T.A.W., R.K., A.E.W., A.C.-L., A.J.P., and G.S.J. The paper was written by A.M.H., J.C.I., V.-A.O., A.J.H., T.A.W., F.J.M.M., A.R.W., J.G., and K.A.F.

DECLARATION OF INTERESTS

The authors declare no competing interests.

Received: February 10, 2019
Revised: June 5, 2019
Accepted: June 24, 2019
Published: July 25, 2019

REFERENCES

1. Verdaguer, D., Jansen, M.A.K., Llorens, L., Morales, L.O., and Neugart, S. (2017). UV-A radiation effects on higher plants: Exploring the known unknown. Plant Sci. *255*, 72–81.
2. Jenkins, G.I. (2017). Photomorphogenic responses to ultraviolet-B light. Plant Cell Environ. *40*, 2544–2557.
3. Cooley, N.M., Higgins, J.T., Holmes, M.G., and Attridge, T.H. (2001). Ecotypic differences in responses of *Arabidopsis thaliana* L. to elevated polychromatic UV-A and UV-B+A radiation in the natural environment: a positive correlation between UV-B+A inhibition and growth rate. J. Photochem. Photobiol. B *60*, 143–150.
4. Hetherington, A.M., and Woodward, F.I. (2003). The role of stomata in sensing and driving environmental change. Nature *424*, 901–908.
5. Isner, J.-C., and Maathuis, F.J.M. (2018). cGMP signalling in plants: from enigma to main stream. Funct. Plant Biol. *45*, 93.
6. DeFalco, T.A., Moeder, W., and Yoshioka, K. (2016). Opening the gates: insights into cyclic nucleotide-gated channel-mediated signaling. Trends Plant Sci. *21*, 903–906.
7. Wang, Y.F., Munemasa, S., Nishimura, N., Ren, H.M., Robert, N., Han, M., Puzõrjova, I., Kollist, H., Lee, S., Mori, I., and Schroeder, J.I. (2013). Identification of cyclic GMP-activated nonselective Ca^{2+}-permeable cation channels and associated *CNGC5* and *CNGC6* genes in Arabidopsis guard cells. Plant Physiol. *163*, 578–590.

8. Cousson, A., and Vavasseur, A. (1998). Putative involvement of cytosolic Ca^{2+} and GTP-binding proteins in cyclic-GMP-mediated induction of stomatal opening by auxin in *Commelina communis* L. Planta *206*, 308–314.
9. Dubovskaya, L.V., Bakakina, Y.S., Kolesneva, E.V., Sodel, D.L., McAinsh, M.R., Hetherington, A.M., and Volotovski, I.D. (2011). cGMP-dependent ABA-induced stomatal closure in the ABA-insensitive Arabidopsis mutant *abi1-1*. New Phytol. *191*, 57–69.
10. Joudoi, T., Shichiri, Y., Kamizono, N., Akaike, T., Sawa, T., Yoshitake, J., Yamada, N., and Iwai, S. (2013). Nitrated cyclic GMP modulates guard cell signaling in Arabidopsis. Plant Cell *25*, 558–571.
11. Isner, J.-C., and Maathuis, F.J.M. (2011). Measurement of cellular cGMP in plant cells and tissues using the endogenous fluorescent reporter FlincG. Plant J. *65*, 329–334.
12. Mülsch, A., Busse, R., Liebau, S., and Förstermann, U. (1988). LY 83583 interferes with the release of endothelium-derived relaxing factor and inhibits soluble guanylate cyclase. J. Pharmacol. Exp. Ther. *247*, 283–288.
13. Beavo, J.A., Rogers, N.L., Crofford, O.B., Baird, C.E., Hardman, J.G., Sutherland, E.W., and Newman, E.V. (1971). Effects of phosphodiesterase inhibitors on cyclic AMP levels and on lipolysis. Ann. N Y Acad. Sci. *185*, 129–136.
14. Kasahara, M., Suetsugu, N., Urano, Y., Yamamoto, C., Ohmori, M., Takada, Y., Okuda, S., Nishiyama, T., Sakayama, H., Kohchi, T., and Takahashi, F. (2016). An adenylyl cyclase with a phosphodiesterase domain in basal plants with a motile sperm system. Sci. Rep. *6*, 39232.
15. Gross, I., and Durner, J. (2016). In search of enzymes with a role in 3′, 5′-cyclic guanosine monophosphate metabolism in plants. Front. Plant Sci. *7*, 576.
16. Bender, A.T., and Beavo, J.A. (2006). Cyclic nucleotide phosphodiesterases: molecular regulation to clinical use. Pharmacol. Rev. *58*, 488–520.
17. Gehring, C., and Turek, I.S. (2017). Cyclic nucleotide monophosphates and their cyclases in plant signaling. Front. Pl. Sci *8*, 1704.
18. Bridges, D., Fraser, M.E., and Moorhead, G.B.G. (2005). Cyclic nucleotide binding proteins in the *Arabidopsis thaliana* and *Oryza sativa* genomes. BMC Bioinformatics *6*, 6.
19. Donaldson, L., Meier, S., and Gehring, C. (2016). The arabidopsis cyclic nucleotide interactome. Cell Commun. Signal. *14*, 10.
20. Beavo, J.A., and Brunton, L.L. (2002). Cyclic nucleotide research – still expanding after half a century. Nat. Rev. Mol. Cell Biol. *3*, 710–718.
21. Jeon, Y.J., Park, S.C., Song, W.S., Kim, O.-H., Oh, B.-C., and Yoon, S.-I. (2016). Structural and biochemical characterization of bacterial YpgQ protein reveals a metal-dependent nucleotide pyrophosphohydrolase. J. Struct. Biol. *195*, 113–122.
22. Matasci, N., Hung, L.H., Yan, Z., Carpenter, E.J., Wickett, N.J., Mirarab, S., Nguyen, N., Warnow, T., Ayyampalayam, S., Barker, M., et al. (2014). Data access for the 1,000 Plants (1KP) project. Gigascience *3*, 17.
23. Puttick, M.N., Morris, J.L., Williams, T.A., Cox, C.J., Edwards, D., Kenrick, P., Pressel, S., Wellman, C.H., Schneider, H., Pisani, D., and Donoghue, P.C.J. (2018). The interrelationships of land plants and the nature of the ancestral embryophyte. Curr. Biol. *28*, 733–745.e2.
24. Morris, J.L., Puttick, M.N., Clark, J.W., Edwards, D., Kenrick, P., Pressel, S., Wellman, C.H., Yang, Z., Schneider, H., and Donoghue, P.C.J. (2018). The timescale of early land plant evolution. Proc. Natl. Acad. Sci. USA *115*, E2274–E2283.
25. Tossi, V., Lamattina, L., Jenkins, G.I., and Cassia, R.O. (2014). Ultraviolet-B-induced stomatal closure in Arabidopsis is regulated by the UV RESISTANCE LOCUS8 photoreceptor in a nitric oxide-dependent mechanism. Plant Physiol. *164*, 2220–2230.
26. Coudert, Y., Palubicki, W., Ljung, K., Novak, O., Leyser, O., and Harrison, C.J. (2015). Three ancient hormonal cues co-ordinate shoot branching in a moss. eLife *4*, e06808.
27. Isner, J.-C., Begum, A., Nuehse, T., Hetherington, A.M., and Maathuis, F.J.M. (2018). KIN7 kinase regulates the vacuolar TPK1 K^+ channel during stomatal closure. Curr. Biol. *28*, 466–472.e4.
28. Favory, J.J., Stec, A., Gruber, H., Rizzini, L., Oravecz, A., Funk, M., Albert, A., Cloix, C., Jenkins, G.I., Oakeley, E.J., et al. (2009). Interaction of COP1 and UVR8 regulates UV-B-induced photomorphogenesis and stress acclimation in *Arabidopsis*. EMBO J. *28*, 591–601.
29. Saidi, Y., Finka, A., Chakhporanian, M., Zrÿd, J.P., Schaefer, D.G., and Goloubinoff, P. (2005). Controlled expression of recombinant proteins in Physcomitrella patens by a conditional heat-shock promoter: a tool for plant research and biotechnology. Plant Mol. Biol. *59*, 697–711.
30. Rymaszewski, W., Dauzat, M., Bédiée, A., Rolland, G., Luchaire, N., Granier, C., Hennig, J., and Vile, D. (2018). Measurement of *Arabidopsis thaliana* plant traits using the PHENOPSIS phenotyping platform. Bio Protoc. *8*, e2739.
31. Sommer, C., Strähle, C., Köthe, U., and Hamprecht, F.A. (2011). ilastik: interactive learning and segmentation toolkit. In Proceedings of the Eighth IEEE International Symposium on Biomedical Imaging (ISBI), pp. 230–233.
32. Eddy, S.R. (2009). A new generation of homology search tools based on probabilistic inference. Genome informatics. International Conference on Genome Informatics *23*, 205–211.
33. Suzek, B.E., Huang, H., McGarvey, P., Mazumder, R., and Wu, C.H. (2007). UniRef: comprehensive and non-redundant UniProt reference clusters. Bioinformatics *23*, 1282–1288.
34. Gough, J., Karplus, K., Hughey, R., and Chothia, C. (2001). Assignment of homology to genome sequences using a library of hidden Markov models that represent all proteins of known structure. J. Mol. Biol. *313*, 903–919.
35. Rice, P., Longden, I., and Bleasby, A. (2000). EMBOSS: the European molecular biology open software suite. Trends in Genetics *16*, 276–277.
36. Edgar, R.C. (2004). MUSCLE: multiple sequence alignment with high accuracy and high throughput. Nucleic Acids Res. *32*, 1792–1797.
37. Altenhoff, A.M., Glover, N.M., Train, C.M., Kaleb, K., Warwick Vesztrocy, A., Dylus, D., de Farias, T.M., Zile, K., Stevenson, C., Long, J., et al. (2018). The OMA orthology database in 2018: retrieving evolutionary relationships among all domains of life through richer web and programmatic interfaces. Nucleic Acids Res. *46* (D1), D477–D485.
38. Katoh, K., and Standley, D.M. (2013). MAFFT multiple sequence alignment software version 7: improvements in performance and usability. Mol. Biol. Evol. *30*, 772–780.
39. Capella-Gutiérrez, S., Silla-Martínez, J.M., and Gabaldón, T. (2009). trimAl: a tool for automated alignment trimming in large-scale phylogenetic analyses. Bioinformatics *25*, 1972–1973.
40. Nguyen, L.T., Schmidt, H.A., von Haeseler, A., and Minh, B.Q. (2015). IQ-TREE: a fast and effective stochastic algorithm for estimating maximum-likelihood phylogenies. Mol. Biol. Evol. *32*, 268–274.
41. Smedley, A.R.D., Rimmer, J.S., Moore, D., Toumi, R., and Webb, A.R.R. (2011). Total ozone and surface UV trends in the United Kingdom: 1979-2008. I. J. Clim. *32*, 338–346.

STAR★METHODS

KEY RESOURCES TABLE

REAGENT or RESOURCE	SOURCE	IDENTIFIER
Bacterial and Virus Strains		
Top10	Fischer scientific (UK)	C404010
BL21(DE3)	Sigma-Aldrich, Merck	CMC0014
Chemicals, Peptides, and Recombinant Proteins		
IBMX	Fischer scientific (UK)	PHZ1124
LY-83,583	Enzo Life Sciences, CAS: 91300-60-6	ALX-550-002-M005
Glutathione Sepharose 4B	(GE Healthcare, Amersham, UK)	GE17-0756-01
Brilliant III Ultra-Fast SYBR qPCR mastermix	Agilent	600882
Pectolyase Y23	Fischer scientific (UK)	ICN320951
Cellulase RS	SERVA Electrophoresis GmbH, Germany	16420
Cellulysin	Calbiochem, Merck, Feltham, UK	219466
BSA	Sigma-Aldrich, Merck	A2153
MES sodium salt	Fischer scientific (UK)	71119-23-8
IPTG	Fischer scientific (UK)	50-121-7428
Critical Commercial Assays		
ELISA cGMP immunodetection	Enzo Life Sciences LTD., Exeter, UK	ADI-900-013
Cyclic Nucleotide Phosphodiesterase assay kit	Enzo Life Sciences LTD., Exeter, UK	BML-AK800-0001
Experimental Models: Organisms/Strains		
Arabidopsis seeds pde1-1	http://arabidopsis.info/	SALK_009905
Arabidopsis seeds pde1-2	http://arabidopsis.info/	SALK_104566
Arabidopsis seeds Columbia-0	Alistair Hetherington lab	N/A
Physcomitrella patens	Jill Harrison's lab [26]	N/A
uvr8-6	Favory et al. (2009) [25]	N/A
Oligonucleotides		
Pde1f	This paper	TGGAAATTGTGGAGCTTGCAG
Pde1r	This paper	TCGTTAGTATCTTCGTCTTCTTGG
act2qrtf	This paper	TTCCAGCAGATGTGGATCTC
act2qrtr	This paper	TCTGTGAACGATTCCTGGAC
pde1GstBamHI_F	This paper	AAAGGATCCATGGCGGCGAAGACGATGA
pde1GstXhoI_R	This paper	AAACTCGAGTCAAGTTGACCCATCCCACT
EF1alpha_F	This paper	TGTGCTGTTCTTATCATTGACTCC
EF1alpha_R	This paper	TGGCATCCATCTTGTTACAACAG
Recombinant DNA		
GST::CN-PDE1	EF1alpha_R	This paper
pGEX6P-1 vector	(GE Healthcare, Amersham, UK)	27-1542-01
Software and Algorithms		
SIMPLE PCI 6.1.2	https://www.hamamatsu.com/	N/A
MXPro 3005 real time PCR system	Agilent	N/A
Prism 6	GraphPad Software	N/A
HMMER3 suite	[27]	N/A
Other		
LEDs 385nm	Ushio Epitex inc., Kyoto, Japan	L385R
LEDs 470nm	Farnell	2414443
LEDs 660nm	Farnell	1142564
LEDs 735nm	Ushio Epitex inc., Kyoto, Japan	L735

LEAD CONTACT AND MATERIALS AVAILABILITY

Further information and requests for resources and reagents should be directed to and will be fulfilled by the Lead Contact, Alistair Hetherington (alistair.hetherington@bristol.ac.uk). Plasmids, *pde1-1* and *pde1-2* knock-outs used in this study are available from the corresponding author on request.

Short-term response to UVA: experimental model and subject details

Plant materials and growth conditions

The ecotype of *Arabidopsis thaliana (L.) Heynh*. used in this study was Columbia-0 (Col-0). The *uvr8-6* mutant is described in Favory et al. (2009) [28]. Plants were grown under short days (10h light:14h dark, 22°C:20°C), 100 μmol m^{-2} s^{-1} photon flux density (PFD), and 70% relative humidity in controlled environment chambers (Snijders, Tilburg, the Netherlands) on a 4:1 mixture of compost and horticultural silver sand. *pde1-1* (SALK_009905) and *pde1-2* (SALK_104566) were obtained from Nottingham *Arabidopsis* Stock Centre (NASC, http://arabidopsis.info/) and knock-out status confirmed by RT-PCR. *Physcomitrella patens* was grown 7-10 days at 24°C with a 16 h: 8 h, light at an irradiance of 100 μmoles.m^{-2}.s^{-1} on BCDAT plates containing 250mg/L $MgSO_4.7H_2O$, 250mg/L KH_2PO_4 (pH6.5), 1010mg/L KNO_3, 12.5mg/L $FeSO_4.7H_2O$, 0.001% Trace Element Solution (TES – 0.614mg/L H_3BO_3, 0.055mg/L $AlK(SO_4)_2.12H_2O$, 0.055mg/L $CuSO_4.5H_2O$, 0.028mg/L KBr, 0.028mg/L LiCl, 0.389mg/L $MnCl_2.4H_2O$, 0.055mg/L $CoCl_2.6H_2O$, 0.055mg/L $ZnSO_4.7H_2O$, 0.028mg/L KI and 0.028mg/L $SnCl_2$.2H2O) and 8g/L agar, supplemented with 1 mM $CaCl_2$ and 1 mM ammonium tartrate) overlaid with a cellophane disk [29]. Protoplasts were isolated from protonemal tissue and transformed as described [26] and transformed with δ-FlincG plasmid using PEG method and the fluorescence was monitored 12h later. Briefly, 7-day old protonemata was added to 1% Driselase and incubated, with gentle agitation for 40 min. Cells were centrifuged and resuspended in MMM medium (0.5 M mannitol, 0.15 M $MgCl_2$, 0.1% MES pH5.6) to achieve a final cell density of 1.5x106 cells mL-1. 10 μg of δ-FlincG plasmid was added to a round bottomed tube. 300 μL of protoplast suspension and 300 μL PEG solution (prepared from 2g polyethylene glycol 6000 dissolved in 5mL of 0.8% mannitol, 0.1M $Ca(NO_3)_2$ 10 mM Tris pH8.0) . The protoplasts were the place for 5 min at 45°C and cooled to 28 degrees for 5 min before being resuspended in 8% mannitol.

METHOD DETAILS

Stomatal aperture measurement

Stomatal movement was studied using epidermal bioassays as described [27]. Abaxial epidermal strips of fully expanded rosette leaves from 5-week-old plants were peeled and floated on 10 mM MES/KOH buffer (pH 6.2) for 30 min to close stomata. The peels were then transferred to opening buffer (10 mM MES/KOH, 50 mM KCl, pH 6.15) and incubated under blue and red (respectively 470 nm and 660 nm, 20nm full width at half maximum) LED panels supplemented with or without UVA LED light (380 nm, FWHM 10nm, Ushio Epitex inc., Kyoto, Japan) from the onset of experimentation or once the stomata were opened depending on the experiment. To test the effect of IBMX on stomatal opening, 50 uM IBMX in DMSO was added in the opening buffer at the start of the experiment and compared with the opening buffer including DMSO as control.

Fluorescence microscopy

Protoplasts were isolated from fully developed leaves, transformed with δ-FlincG and visualized using an epifluorescence microscope (Diaphot-TMD; Nikon, https://www.nikon.com/) with a 60x oil immersion objective. cGMP-dependent fluorescence was recorded every 20 s using 480/20-nm excitation and 520/40-nm emission. Fluorescence images were acquired with a Hamamatsu 1394 ORCA-ERA digital camera (Hamamatsu, https://www.hamamatsu.com/), and analyzed with SIMPLE PCI 6.1.2 offline imaging software (Compix Imaging Systems, https://hcimage.com).

ELISA cGMP immunodetection

An ELISA assay kit (cGMP Activity Assay kit; Biovision, https://www.biovision.com/) was used to measure the concentration of cGMP. *Arabidopsis* at rosette stage were illuminated with or without UVA LED lights for 0, 5 and 20 min before freezing. Frozen tissue was ground with a mortar and pestle in 0.1 M HCl, and the disrupted tissue was filtered through four layers of Miracloth. cGMP was extracted from the filtrate with 0.1 M HCl, freeze-dried and re-dissolved in 0.1 mL of 0.1 M HCl. The acetylation and quantification of cGMP by ELISA was carried out according to the protocol provided by the manufacturer.

In vitro activity of PDE1

PDE1 was amplified from mRNA extracted from leaves with pde1GstBamHI_F and pde1GstXhoI_R primers (Table S1) and cloned into pGEX6P-1 vector (GE Healthcare, Amersham, UK). GST and GST::PDE1 expression was induced in 1l culture of *E. coli* BL21 at 37°C with 1mM IPTG for 4 hours. Cells were collected and lysed in TBS-T buffer (TBS pH 7.3, 0.1% Trion X-100) by sonication. After centrifugation, the lysate was cleared using 0.45 um filter. GST or GST::PDE1 was bound to 500 μl of Glutathione Sepharose 4B (GE Healthcare, Amersham, UK) for 2h and eluted with 10mM 50mM TRIS-HCl 10mM GSH reduced pH 8.0. Cyclic Nucleotide Phosphodiesterase assay was performed according to the Cyclic Nucleotide Phosphodiesterase assay kit (Enzo Life Sciences LTD., Exeter, UK). Briefly, purified GST::PDE1 and GST were desalted using the column provided and the effective removal of phosphate and nucleotides from the extract was tested qualitatively using the Biomol® Green reagent provided. All of the enzyme assays were conducted at 25°C for 60 min.

qPCR

Guard cell protoplasts were isolated according to Isner et al. [27]. Briefly, 40 fully expanded leaves were blended in water for 1 min using a waring SS515 Blender (Cole-Parmer UK) and poured onto a 200 μM mesh to collect epidermes. Epidermes were incubated in 45% H2O, 55% basic solution (0.5 mM $CaCl_2$, 0.5mM $MgCl_2$, 0.01 mM KH_2PO_4, 0.5 mM ascorbic acid, 550 mM sorbitol, 0.2% BSA, 0.7% cellulysin, 5 mM MES/Tris pH 5.5) for 1.5 h at 30°C. The epidermes were then incubated in basic solution supplemented with cellulase Onozuka RS 0.01% and pectolyase Y23 for 1 h at 30°C. Guard cell protoplasts were collected after passing the solution through a 20 μm mesh. RNA was extracted using the Machery-Nagel Nucleospin II plant RNA extraction kit incorporating DNase I treatment (Thermo Fisher Scientific). cDNA was synthesized using the High Capacity cDNA Reverse Transcription Kit with RNase Inhibitor (Applied Biosystems), according to the manufacturer's instructions with hexamers. cDNA was analyzed using an MXPro 3005 real time PCR system (Agilent) with Brilliant III Ultra-Fast SYBR qPCR mastermix (Agilent) using the primer Pde1f+r, and act2qrtf+r primers designed such that one of the primer pair sequence spans an intron (Table S1). The amplified products were then loaded on an agarose gel and imaged under UV light. At least 3 technical repeats were performed for each qRT-PCR reaction. Data were analyzed using the ΔΔCt method, with ACT2 as a reference transcript.

Long-term response to UVA experiment

Growth conditions

Col-0, *pde1-1* and *pde1-2* plants were grown in a growth chamber in individual 225 mL culture pots containing a 30:70 (v/v) mixture of a loamy soil and organic compost (Klasmann). Plants were cultivated with a daily cycle of 12 h light supplied from a bank of fluorescent light tubes (5 FHO Luxline Plus, Sylvania) complemented with a set of blue and red LEDs as described in the short-term experiment (respectively λmax = 470 nm and λmax = 660 nm), with or without UVA (370 nm, FWHM 10 nm). The overall photosynthetic photon flux density (PPFD) at plant height was 120 μmol m^{-2} s^{-1}, and the pots were moved daily to avoid boundary effects. Temperature and relative humidity were constantly monitored and maintained identical for both –UVA and +UVA scenarios (22/20°C day/night, 70% air relative humidity). Pots were sealed to prevent water evaporation from the soil. From germination to bolting, each pot was weighed daily and watered at a target weight to maintain soil water content at 1.5 g H_2O g^{-1} dry soil, previously determined as a well-watered level non-limiting for growth [30].

Growth, transpiration and water-use efficiency measurements

Daily estimates of projected rosette area were calculated for each plant using top images taken every two days in the cabinet and analyzed with Ilastik software, version 1.21.7 [31]. Briefly, total pixels corresponding to plants were extracted from background using a random forest classification method based on color and texture and later converted to mm. Plants were harvested at bolting (corresponding to ca. 6 weeks after sowing for all 3 genotypes), their dry weight was measured and they were dissected to measure their final, total leaf area. From germination to bolting, cumulated water loss through transpiration was determined by daily weighing each sealed pot. Whole plant water-use efficiency (WUE) was then computed as the ratio between plant final dry weight and the total amount of transpired water over the experiment duration. Transpiration rate during the daytime was determined 5 days before harvest. Each pot was weighed with 10^{-3} g accuracy (Precisa balances XB, Precisa) at the beginning and end of the day (light) period. Weight losses over 12 h light period were used to calculate average daytime transpiration rate on a leaf area basis (E), using the projected rosette area estimated the same day for each plant. Final dry weight, final total leaf area, WUE and E were measured on 8 plants per genotype in each scenario (-UVA or +UVA).

qPCR analysis of PDE1 *transcript abundance in response to UVA*

To assess whether *PDE1* transcript abundance is affected by UVA, RNA was extracted from the rosette leaves of 5 week old Col-0 plants 6h post-dawn (midday) using the Nucleospin® RNA extraction kit (Machery-Nagel). RNA was extracted from plants grown without UVA, and with UVA, or plants that had been grown under no UVA light and transferred to UVA light 3 hours before sampling. RNA was converted to cDNA using the High Capacity cDNA Reverse Transcription Kit (Applied Biosystems). QPCR was carried out using the Brilliant III Ultra-Fast SYBR® Green QPCR kit on a Stratagene Mx3005P real time PCR system (Agilent Technologies). Three biological replicates were performed for each treatment and three technical replicates were performed for each QPCR reaction. Transcript abundance was calculated with the ΔΔCt method using *EF1α* as a reference transcript. Data were analyzed using a one-way ANOVA.

Bioinformatic analysis

Five sets of reference sequences were produced from proteins with potentially relevant molecular function, expressed in other organisms (Data S1). Jackhmmer from the HMMER3 suite [32] was used to build profile hidden Markov model (HMM) libraries against *uniref90* [33] for all amino acid sequences of the human PDEs that hydrolyse cGMP (families 1, 2, 3, 5, 6, 9, 10, and 11). The libraries were searched using hmmsearch from HMMER3, against all plant proteins in the SUPERFAMILY database [34]. The catalytic domains of the 2 best scoring search hits and their human seeds were truncated and used to create the first set of reference sequences. The hits were also filtered for proteins expressed in *Arabidopsis thaliana* and examined for cnPDE candidates.

The SUPERFAMILY database was also used to identify plant proteins containing a domain with a high confidence (*e-value* < 0.0001) PDEase family assignment and closest in structure to a SCOP domain with cGMP hydrolysing function. Only results from the following 3 genomes were retained, due to being sequenced at a higher quality than the rest: *Micromonas pusilla CCMP1545 v3.0*, *Volvox carteri f. nagariensis* and *Chlamydomonas reinhardtii 4.0*. The 3 proteins with the most confident PDEase family assignment were used as the second set of reference sequences. A third set of domain-only sequences was created from truncating the PDEase domain of each

of the 3 proteins. Finally, the SUPERFAMILY database was queried for plant proteins with a domain assigned to the superfamily HD classification with high confidence (*e-value* < 0.0001) and with low confidence (*e-value* > 0.0001) to a non-PDEase family, or which had no family classification. 100 results were retained which had the most confident classification at the superfamily level. Of those, the 10 sequences with the least confident classification at the family level and which were at least 200 amino acids long were used to create a fourth set of reference sequences. As before, the domains were truncated to create a fifth and final reference set.

Using jackhmmer, profile HMMs were then built against *uniref90* for every reference sequence in the 5 sets. The profile HMM libraries were searched using hmmsearch, against the 6 frames of translation in *Arabidopsis thaliana* (constructed by running sixpack from EMBOSS [35] on the nucleic acid sequence assembled by NCBI from TAIR10). Profile HMMs from reference set 1 were also searched against all proteins expressed in *Arabidopsis thaliana,* retrieved from SUPERFAMILY. To ascertain the significance of search results, high-scoring matches and their corresponding seed sequences were manually examined, to identify whether the catalytic domain was hit, how well the two sequences aligned, using MUSCLE [36], and where each result mapped on the *Arabidopsis thaliana* genome.

Phylogenomic analysis

Orthologs of AtPDE1 from across the tree of life were identified from the OMA database [37]. This ortholog set was supplemented with AtPDE1 homologs identified using BLASTP searches against an additional set of genomes chosen to help clarify the distribution of the gene family in non-model eukaryotes, and to determine the origin of the eukaryotic gene (whether from the eukaryotic nuclear lineage, or from the mitochondrial or plastid endosymbionts). Sequences were aligned using the most accurate l-INS-i mode in mafft [38], poorly aligning positions were identified and filtered out using the "gappyout" mode in trimAl [39], and a bootstrapped maximum likelihood phylogeny was inferred using IQ-Tree 1.6.5 [40] under the LG+C60+F model, which was the best-fitting substitution model according to the both BIC and AIC model selection criteria.

UVA spectrum

The UV-visible solar spectrum (total (direct+diffuse) radiation on a horizontal surface) is monitored on a routine basis at a site in Reading, UK (51.44N, 0.94W) using a Bentham DM150 spectroradiometer with calibration traceable to NIST, USA. Details of the site, calibration and quality assurance are given in [41] and the site is a Global Atmospheric Watch Regional station for ozone and UV radiation. Spectra are measured every 30 min from 290 – 500 nm at 0.5 nm resolution. A number of cloudless days were identified from ancillary data and each spectrum for the day divided into UVB (290 – 315 nm) UVA (315 – 400 nm) and Blue (400-500 nm) wavebands. The spectral irradiance was integrated across each waveband to give UVB, UVA and Blue irradiance. The ratios UVB/UVA, UVA/Blue and UVB/Blue were computed for each time point to illustrate the diurnal variation in each waveband ratio.

QUANTIFICATION AND STATISTICAL ANALYSES

Statistical tests

Unless stated otherwise, unpaired Student's t test was used to compare 2 samples and ANOVA was used when with Tukey post hoc analysis if more than 2 samples were compared. Shapiro-Wilk test was used to test the normality and the homoscedasticity of the residuals was assessed by calculating the variance of the different samples. Letters denotes a significance with a $p < 0.05$. Other details are to be found in the legends.

DATA AND CODE AVAILABILITY

Bioinformatics data are available in Data S1.

Current Biology, Volume *29*

Supplemental Information

Short- and Long-Term Effects of UVA on *Arabidopsis* Are Mediated by a Novel cGMP Phosphodiesterase

Jean-Charles Isner, Vlad-Aris Olteanu, Alexander J. Hetherington, Aude Coupel-Ledru, Peng Sun, Ashley J. Pridgeon, Glyndyr S. Jones, Matthew Oates, Tom A. Williams, Frans J.M. Maathuis, Richard Kift, Ann R. Webb, Julian Gough, Keara A. Franklin, and Alistair M. Hetherington

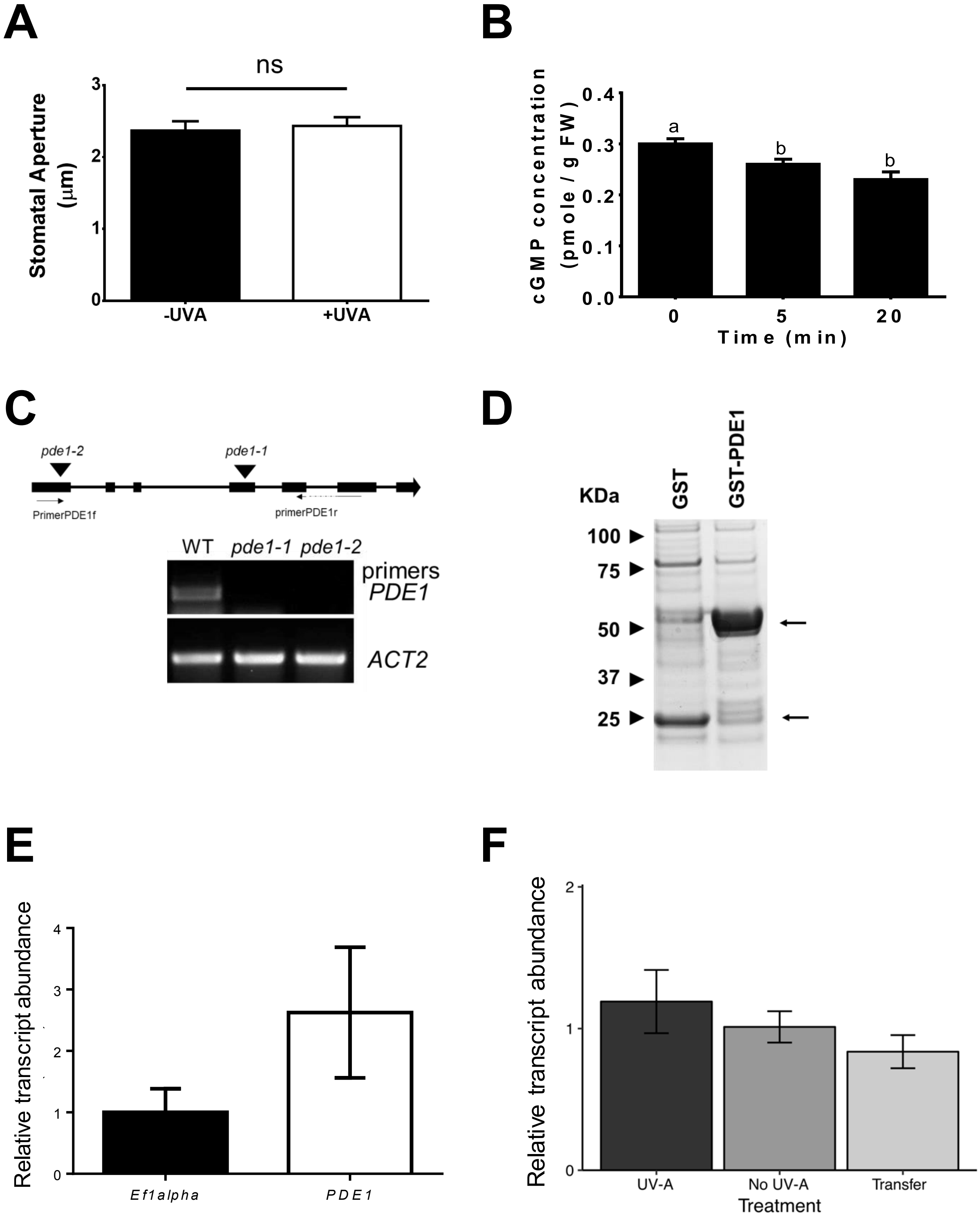
A
ns
Stomatal Aperture (µm)
3
2
1
0
-UVA
+UVA
B
cGMP concentration (pmole / g FW)
0.4
0.3
0.2
0.1
0.0
a
b
b
0
5
20
Time (min)
C
pde1-2
pde1-1
PrimerPDE1f
primerPDE1r
WT
pde1-1
pde1-2
primers
PDE1
ACT2
D
KDa
GST
GST-PDE1
100
75
50
37
25
E
Relative transcript abundance
4
3
2
1
0
Ef1alpha
PDE1
F
Relative transcript abundance
2
1
0
UV-A
No UV-A
Transfer
Treatment

Figure S1: Characterisation of UVA effects on cGMP signaling pathway. Related to Figures 1, 2 and 3.

(A) UV-A radiation does not induce stomatal closure.

Isolated Arabidopsis epidermis was incubated in 10 mM MES/KOH, 50 mM KCl, pH 6.15 and illuminated with 10 µmol m^{-2} s^{-1} blue (λmax=470 nm) and 50 µmol m^{-2} s^{-1} red (λ=660 nm) for 3 hours. Subsequently, 10 µmol m^{-2} s^{-1} UV-A (λmax=380 nm) was added, or not, together with blue/red light for another 3h when stomatal apertures were measured. Data represent means (+/- SEM) of 90 stomatal aperture measurements from three replicates. Statistical analyses were performed by non-paired Student's t-test. No significant (ns) difference was observed ($p > 0.05$).

(B) UV-A induces a reduction in cGMP. Arabidopsis leaves (rosette stage) were illuminated with or without UV-A LED lights for 0, 5 and 20 min and cGMP content was determined by ELISA Exposure to UV-A (10 µmol m^{-2} s^{-1} (λmax=380 nm) results in a decrease of cGMP after 5 and 20 min. Data represent means (+/- SEM) of three replicates. Statistical analyses were performed by one-way ANOVA with Tukey posthoc analysis, and letters show significant differences at $p < 0.05$.

(C) Characterisation of cn*pde1* KO mutants. A. Exon-intron gene diagram of *AtCN-PDE1*. Exons are in black boxes. B. PDE1 was amplified by RT-PCR using gene specific primers (primerPde1f and primerPde1r, see Figure S3A) in WT. The absence of PDE1 amplification demonstrates that *pde1-1* and *pde1-2* are KO due to the presence of T-DNA. C. Sequences and domain architecture of 26 candidate PDE proteins in Arabidopsis. At1G17330 was the only one of these candidates to show a UV-A phenotype upon knockout.

(D) *In vitro* expression of GST-PDE1 in *E. coli*. *PDE1* was inserted in pGEX-6P vector. pGEX-PDE1 or empty vector was transformed into *E.coli* BL21. Expression was induced with 1mM IPTG for 4 hours at 37°C. GST-PDE1 or GST was purified using Sepharose 4G and loaded on a denaturating acrylamide gel. Gel was stained with InstantBlue™. GST-PDE1 and GST are shown by arrows at ~26KDa and 51KDa.

(E) *PDE1* is expressed in guard cells and its abundance is not affected by UV-A treatment in leaf tissue. Guard cell protoplasts were extracted from Arabidopsis leaves (see material and methods). qPCR was performed on cDNA synthetized on the RNA extracted from the guard cell protoplasts using pde1f+r primers. CN-PDE1 transcript was compared with EF1α (n=3).

(F) The transcript abundance of *PDE1* is not affected by long term or short term exposure to UV-A in leaf tissue. QPCR was performed on rosette leaves of 5 week old *Arabidopsis* Col-0 leaves that were grown under UV-A (UVA), under no UV-A (No UVA), or plants that had been grown under no UV-A and then treated with UV-A for 3 hours (Transfer). *EF1α* was used as a reference transcript. Data show the mean ± SEM ($n = 3$). No significant differences between the treatments was observed when analysed using a one-way ANOVA.

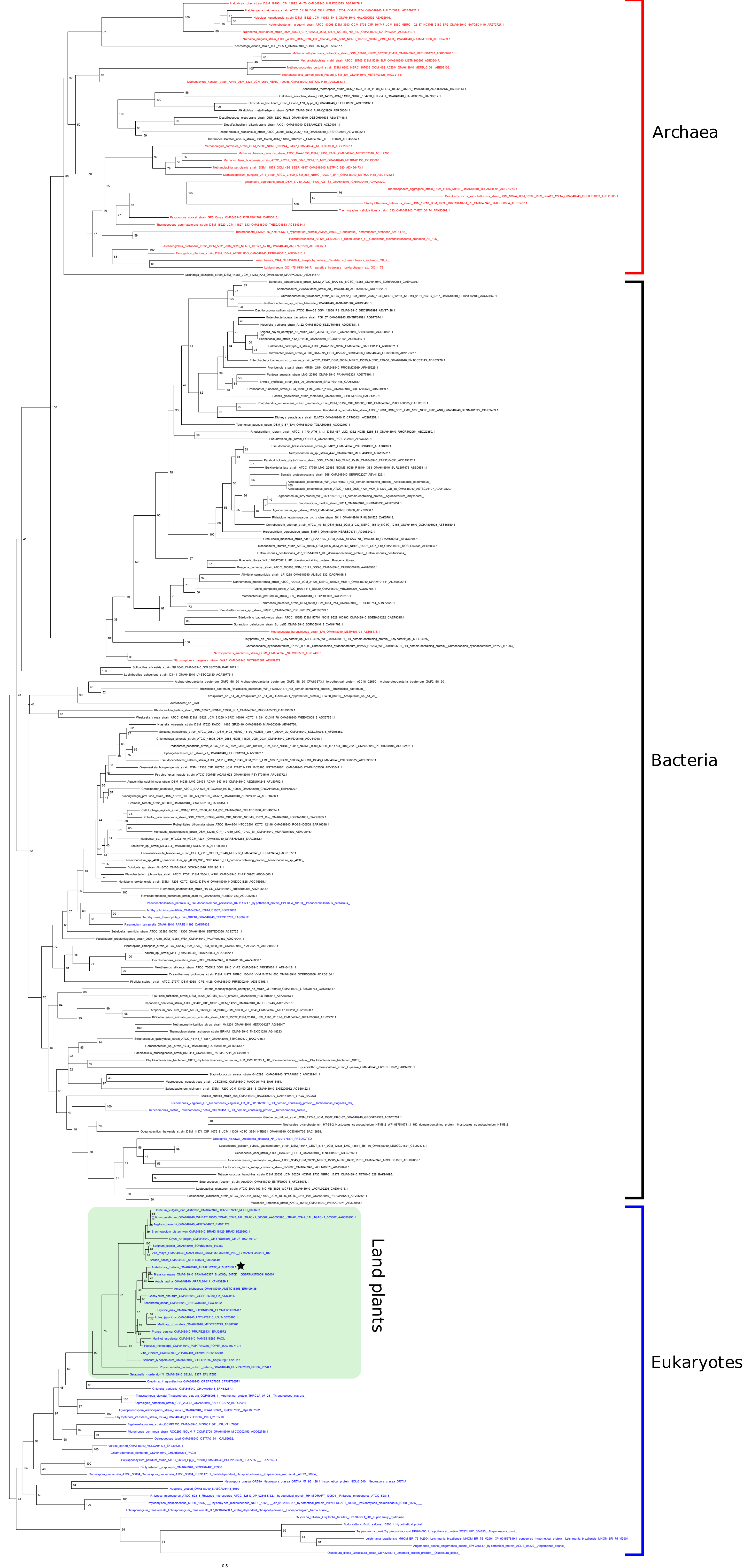
Archaea
Bacteria
Land plants
Eukaryotes
0.5

Figure S2: *CN-PDE* Tree. Related to Figure 4.

AtCN-PDE1 is part of a gene family that is conserved across the tree of life but appears to have been specifically lost in the animal lineage. Most of the eukaryotic sequences - including those from nonphotosynthetic lineages - form a monophyletic group, suggesting a single origin in the last eukaryotic common ancestor. The genes from two ciliates, *Tetrahymena* and *Paramecium*, and the excavates *Trichomonas vaginalis* and *Trichomonas foetus* appear to be recent horizontal gene acquisitions from Bacteria. The eukaryotic clade groups with Bacteria in the tree, and although members of the Asgard archaea – the closest archaeal relatives of eukaryotes – encode members of the AtPDE family, these genes are not closely related to those of eukaryotes. The phylogeny was inferred under the LG+C60+F model in IQ-Tree; branch lengths are proportional to the expected number of substitutions per site. Archaea sequences red, bacteria sequences black, eukaryotic sequences blue. Land plants highlighted with green box. At1G17330 (*CN-PDE1*) highlighted with black star.

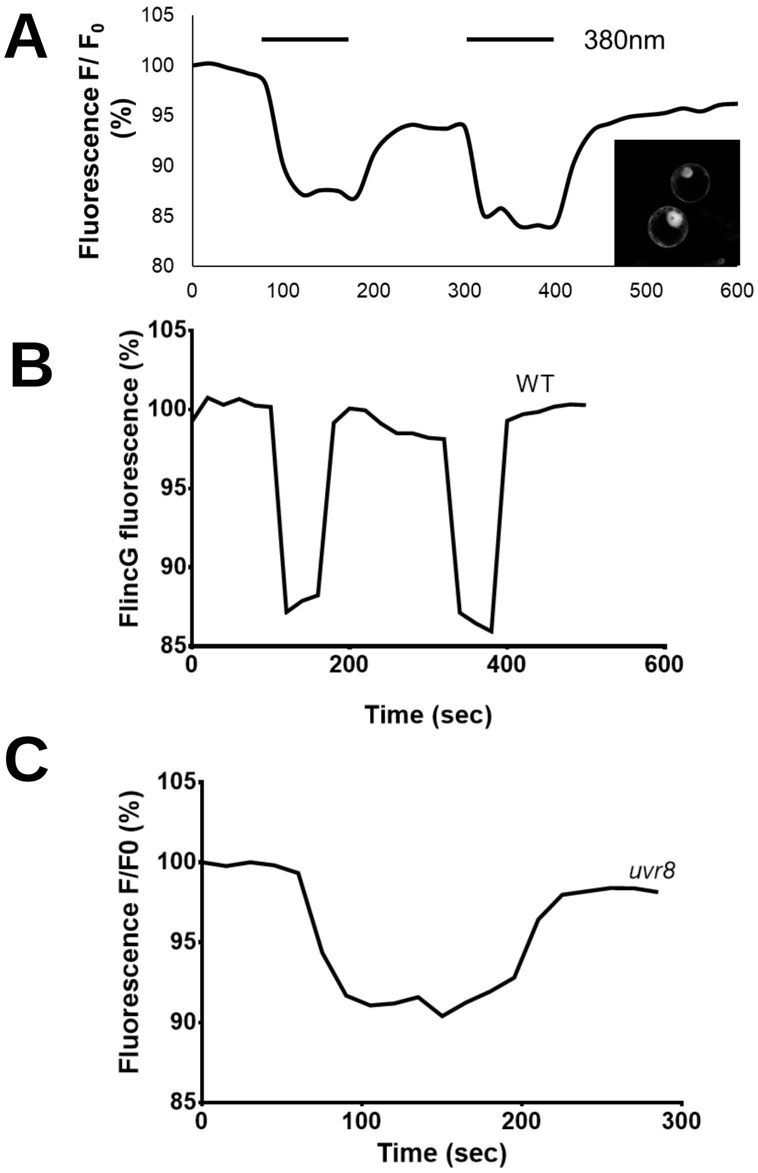

Figure S3. The UV-A signalling pathway is conserved in *Physcomitrella patens* (A) and does not involve UVR8. Related to Figure 1.

(A) Protoplasts of the moss *Physcomitrella patens* were isolated from protonemal tissue and transformed with δ-FlincG plasmid. Protoplasts were incubated 12h to allow δ-FlincG expression and the effect of UV-A on the fluorescence was monitored as described previously. The speed and the intensity of the decrease of δ-FlincG fluorescence upon UV-A illumination was similar to that observed in Arabidopsis. The graph shown is representative of 5 experiments.

(B-C) *uvr8* exhibits wild type UV-A-induced reduction in δ-FlincG fluorescence. Mesophyll protoplasts extracted from *uvr8* were transiently transformed with δ-FlincG plasmid. Protoplasts were incubated 12h to allow δ-FlincG expression and the effect of UVA on the fluorescence was monitored as described previously See methods). *uvr8* (C) showed a similar response to the WT (B) when illuminated with UVA (10 µmol m^{-2} s^{-1} (λmax=380 nm). A representative graph is shown for *uvr8* mutant tested (n > 5).

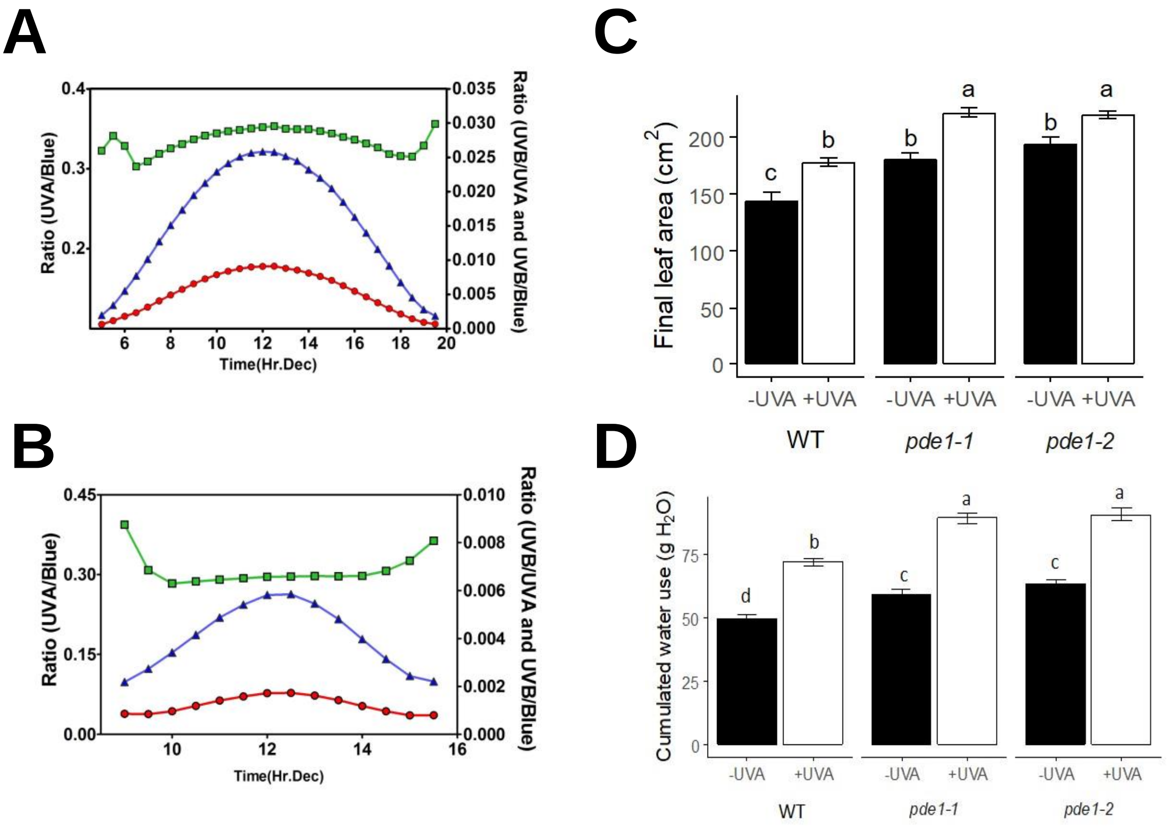

Figure S4. Diurnal cycle of UV and Blue waveband ratios in the solar spectrum and long-term UV-A effects on growth and cumulated water loss. Related to Figure 4.

(A-B) Ratios of UV-A/Blue (green squares), UV-B/Blue (red circles) and UV-B/UV-A (blue triangles) wavebands of solar radiation for cloud-free days. Measurements were made at Reading, UK (51.5N) in summer (24/7/2012) (A) and winter (13/12/2014) (B) when there were minimal disturbances due to changes in the atmosphere. Similar diurnal patterns are observed on other cloud-free days. The wavebands are the unweighted integrals of spectral measurements: UV-B = 280-315 nm; UV-A = 315-400 nm; Blue = 400-500 nm. (C-D) Long-term UV-A exposure increases growth and cumulated water loss. Arabidopsis plants were grown under light tubes supplemented with a combination of blue (λmax=470 nm) and red (λmax=660 nm) light coupled or not to UV-A light (λmax=370 nm) (as in Fig 1). For each plant, final, total plant rosette area was measured at harvest (bolting) after dissecting the plant (A). Individual pots were sealed to prevent water evaporation from the soil. From germination to bolting, cumulated water loss through transpiration was determined by daily weighing each sealed pot. The total amount of transpired water over the experiment duration is represented in (B). Data represent means (+/- SE) of 8 plants per genotype and light scenario. Statistical analyses were performed by one-way ANOVA with Tukey post-hoc analysis, and letters show significant differences at $p < 0.05$.

**Primer Name**	**Sequence**
PDE1 QPCR FP	GAAGGCGATGAAAGGAAACGA
PDE1 QPCR RP	CTGCAAGCTCCACAATTTCC
primerPde1f	TGGAAATTGTGGAGCTTGCAG
primerPde1r:	TCGTTAGTATCTTCGTCTTCTTGG
EF1α QPCR FP	TGTGCTGTTCTTATCATTGACTCC
EF1α QPCR RP	TGGCATCCATCTTGTTACAACAG
pde1GstBamHI_F	AAAGGATCCATGGCGGCGAAGACGATGA
pde1GstXhoI_R	AAACTCGAGTCAAGTTGACCCATCCCACT

Table S1. Primer Sequences. Related to STAR Methods.
